# Origin Determination of Walnuts (*Juglans regia* L.) on a Worldwide and Regional Level by Inductively Coupled Plasma Mass Spectrometry and Chemometrics

**DOI:** 10.3390/foods9111708

**Published:** 2020-11-20

**Authors:** Torben Segelke, Kristian von Wuthenau, Anita Kuschnereit, Marie-Sophie Müller, Markus Fischer

**Affiliations:** Hamburg School of Food Science, Institute of Food Chemistry, University of Hamburg, Grindelallee 117, 20146 Hamburg, Germany; torben.segelke@chemie.uni-hamburg.de (T.S.); kristian.wuthenau@chemie.uni-hamburg.de (K.v.W.); anita.kuschnereit@studium.uni-hamburg.de (A.K.); marie-sophie.mueller@studium.uni-hamburg.de (M.-S.M.)

**Keywords:** walnut, *Juglans regia*, origin authentication, element profiling, inductively coupled plasma mass spectrometry, ICP-MS, chemometrics, isotopolomics

## Abstract

To counteract food fraud, this study aimed at the differentiation of walnuts on a global and regional level using an isotopolomics approach. Thus, the multi-elemental profiles of 237 walnut samples from ten countries and three years of harvest were analyzed with inductively coupled plasma mass spectrometry (ICP-MS), and the resulting element profiles were evaluated with chemometrics. Using support vector machine (SVM) for classification, validated by stratified nested cross validation, a prediction accuracy of 73% could be achieved. Leave-one-out cross validation was also applied for comparison and led to less satisfactory results because of the higher variations in sensitivity for distinct classes. Prediction was still possible using only elemental ratios instead of the absolute element concentrations; consequently, a drying step is not mandatory. In addition, the isotopolomics approach provided the classification of walnut samples on a regional level in France, Germany, and Italy, with accuracies of 91%, 77%, and 94%, respectively. The ratio of the model’s accuracy to a random sample distribution was calculated, providing a new parameter with which to evaluate and compare the performance of classification models. The walnut cultivar and harvest year had no observable influence on the origin differentiation. Our results show the high potential of element profiling for the origin authentication of walnuts.

## 1. Introduction

Walnuts are the seeds of the *Juglans* tree, particularly of the English or Persian walnut tree *Juglans regia*. They are appreciated for their high level of polyunsaturated fatty acids as well as their high tocopherol and potassium content. There are different walnut cultivars that are commercially grown—e.g., “Lara” and “Chandler” [1,2,3]. Nowadays, consumers are increasingly interested in products made with selected ingredients. The interest in sustainable and regional food is growing correspondingly. As a result, consumers are willing to accept higher prices for products with a specific geographical origin [4]. The annual financial damage by food fraud is estimated at 40 billion dollars, and there are also health risks with lethal consequences [5]. The omics disciplines are suitable for authentication by creating a fingerprint of the examined food to prevent food fraud [4]. DNA-based methods for food authentication, which are only able to determine the genotype, are inevitably limited to the determination of the biological identity [6,7]. Only in exceptional cases where only certain varieties are grown in certain regions can indications of geographical origin be obtained. Furthermore, the presence of the analyte DNA is the essential prerequisite for carrying out molecular biological analyses. This is usually not the case with fats and oils [4,8].

Isotopolomics is particularly applicable for origin analysis, as it reflects the influence of the soil and thus the geographical origin [9,10]. Inductively coupled plasma mass spectrometry (ICP-MS) has become a routine application in the field of isotopolomics for the generation of the elemental profiles of food by the quantitative determination of the elemental composition of the sample in a wide dynamic range (ng/kg to mg/kg) [11,12,13,14].

Walnuts have been analyzed before with regard to their origin, partly by determining the elemental content. Esteki et al. used chromatographic fatty acid fingerprint analysis to differentiate walnuts from six Iranian regions [15]. Gu et al. analyzed Chinese walnut samples with inductively coupled plasma optical emission spectrometry and near-infrared and mid-infrared spectroscopy from three production areas in Xinjiang [16]. Krauß et al. evaluated stable isotope signatures from different regions in Germany [17]. In a preliminary but international study by Popescu et al., the authors used nuclear magnetic resonance spectroscopy to investigate the differences between walnuts varieties from five countries and two years of harvest [18].

To our knowledge, however, there has been no large-scale international walnut study based on the analysis of elementary patterns comparing several geographical origins from at least three harvesting years [19]. The element pattern is considered particularly suitable for determining the geographical origin of walnuts, since walnut kernels grow inside the shell and are therefore protected from the environment—i.e., practically unaffected by perturbations such as anthropogenic aerosols and soil dust [2,20]. As a consequence, the exclusive elemental characteristics of the soil should be recognized in the walnut kernels. Still, the analysis may be challenging from an analytical point of view, since only small quantities of elements are to be expected in the fat-rich walnut kernels [20], requiring sensitive analytical methods. ICP-MS offers limits of detection into the parts per trillion (ng/L) range [10,13], and is therefore even more sensitive than inductively coupled plasma optical emission spectrometry (ICP-OES), as applied in previous analyses of walnuts [3,21,22,23].

Therefore, the aim of this study was to develop a reliable chemometric model using ICP-MS in combination with machine learning methods for the worldwide and regional origin authentication of walnuts, independent from harvest year and cultivar.

In this context, we focused on the *Juglans regia* walnut species, as it is grown and consumed all over the world and is generally considered to be of the highest quality and has the highest demand [15,17].

In 2018, about 3.6 million tons of walnuts were harvested. China (approx. 1.6 million tons) and the USA (approx. 0.6 million tons) have the largest contribution to the worldwide walnut harvest. In the context of walnut authentication for the west European market, however, not only is the total harvest quantity important but also the quantity of exported and imported goods. Chile, for example, is a significant contributor, exporting more than 10,000 tons to Germany, France, Italy, and Switzerland combined. Turkey, Hungary, and Pakistan also play an important role [1,17,24].

Consequently, 237 walnut reference samples from three harvest years and originating from ten countries were analyzed with high-resolution ICP-MS. Principal component analysis (PCA) [25] and *t*‑distributed stochastic neighbor embedding (*t*-SNE) [26] were carried out to visualize the data. Then, machine learning methods were applied to develop classification models for the authentication of walnuts from ten countries on a worldwide scale. Since a high number of authentic walnut samples (>30) could be obtained from France, Germany, and Italy, classification models were also developed on a regional level.

## 2. Materials and Methods

### 2.1. Reagents and Materials

The elemental analyses of walnuts were based on a previous study [13]. In Table S1 (supporting information), the reagents and materials used in this study are listed.

### 2.2. Sample Preparation

**Figure 1:**
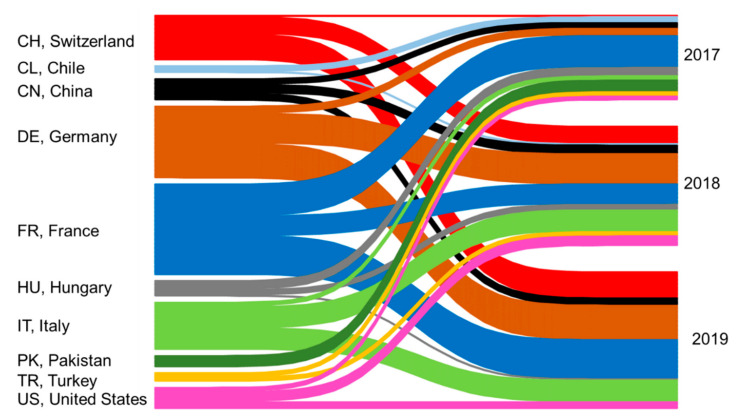
Overview of the 237 walnut samples with regard to their origin and harvest year.

A total of 237 reference samples of relevant, market-available walnuts from three years of harvest (2017, 2018, and 2019) were collected and analyzed in this study. The walnut samples originated from ten countries and were purchased as shelled or in-shell goods. Thanks to the cooperation with regional producers and project partners who work according to our internal guidelines to ensure the authenticity of the reference material (e.g., by applying the HACCP guidelines, FSSC 22,000, or providing the structure meta date), authentic walnut samples could be acquired. See Table S2 (Supporting Information) for detailed information and Figure 1 for a visual illustration. On arrival, walnut samples were frozen and stored at −80 °C until further processing could take place. Element patterns are less influenced by storage than other profiling levels, such as the metabolome [27]. At the applied storage conditions of −80 °C, enzyme activities are inhibited—i.e., cell lysis is also inhibited [28]. One German walnut sample (harvest year 2018, Hesse) was selected as a quality control (QC) sample.

### 2.3. Sample Preparation and Digestion

For each walnut sample, 100 g of walnut kernels were milled using a knife mill (Grindomix GM 300, Retsch, Haan, Germany) with the addition of dry ice. If necessary, in-shell walnuts were shelled before. Homogenized samples were freeze-dried for 48 h (Beta 1–8 LDplus, Martin Christ Gefriertrocknungsanlagen GmbH, Osterode am Harz, Germany), including a stirring step after 24 h.

The sample digestion of 500 mg of homogenized and lyophilized walnut material was performed using an Ethos.lab microwave (MLS GmbH, Leutkirch, Germany), as described in reference [13] and in Table S3 (Supporting Information). For each digestion run, one vessel was selected for the QC sample and one vessel for a blank. The QC sample was later used for quality assurance and the calculation of the method’s precision (see Section 3.2).

### 2.4. Analytical Procedure and Instrumentation

Multi-elemental analyses were performed on an HR-ICP-MS Element2 (ThermoFisher Inc., Waltham, MA, USA) coupled with an SC-E4 Autosampler (Elemental Scientific Inc., Omaha, NE, USA), following a method validated in a previous study [13].

The multi-element method included 47 isotopes: Li, Be, B, Na, Mg, Al, K, Ca, Sc, V, Cr, Mn, Fe, Co, Ni, Cu, Zn, Ga, As, Se, Rb, Sr, Y, Mo, Ag, Cd, Te, Ba, La, Ce, Pr, Nd, Sm, Eu, Gd, Tb, Dy, Ho, Er, Tm, Yb, Lu, Tl, Pb, Bi, Th, U. The calculated limit of detection and limit of quantitation are given in Table S4 (Supporting Information). Here, also the respective element used for internal standardization is given.

Quantitation was conducted by external calibration. Instrument optimization, mass calibration, and mass offset were performed daily. For further instrumental conditions and method validation, refer to Table S5 (Supplementary Materials) and reference no. [13]. Tubes and pipette tips were pre-cleaned by soaking in 3% (*v*/*v*) nitric acid overnight and subsequently rinsed with ultrapure water and dried.

### 2.5. Multivariate Data Analysis and Classification Models

Multi-element data were visualized and interpreted with one-way analysis of variance [29] (ANOVA) tests using Matlab R2019a (The Mathworks Inc., Natick, MA, USA). Bonferroni post-hoc tests [30] were calculated to determine inter-class differences using Microsoft Excel 2016 (Microsoft Corporation, Redmond, WA, USA).

Boxplots for the data visualization of certain elements, including outlier detection, were created using Matlab’s boxplot-function [31]. Furthermore, *t*-SNE (Barnes–Hut algorithm, cosine distance, perplexity = 18) and PCA plots were calculated using Matlab. For interpretation, 95% confidence ellipses were added to the score plots [32].

For the calculation of classification models, Matlab was applied; different settings of the data pre-treatment, classification method, and validation were compared for each sub-issue (the differentiation of all walnuts or the differentiation of only French walnuts, etc., as described in the following sections). The settings for the data pre-treatments, the classification methods, and the validation are stated in Table 1. For classification methods, we chose linear discriminant analysis, support vector machine [33], subspace discriminant [34,35], and random forest [36]. Following a design of experiments approach, the results of every combination were compared and the settings of the best results were chosen [37].

For obtaining an unbiased estimate of the model’s performance, the models were validated using (a) leave-one-out cross validation or (b) stratified nested cross validation [38,39]. Since the former validation method is more widespread in the scientific community, we would like to explain the procedure of the latter validation method briefly: The whole data set was split into five parts, whereby the samples were not fully randomly divided but stratified by the origins. Hence, all five parts contained a preferably equal number of samples with respect to the walnuts’ origins, ensuring a representative and balanced training (four fifth) and test (one fifth) set. For the training set, 10-fold cross validation was applied to select the optimal model parameters (i.e., inner cross validation). The performance of the calculated model was evaluated by predicting the independent test set. The described procedure was repeated for all five parts, so every part of the 5-fold outer cross validation was once used as the test set (i.e., outer cross validation). Finally, since the results by a single nested cross validation can vary, the entire cross validation was repeated 20 times. By repeating this process, a standard deviation of the accuracy was calculated [13].

For the geographical origin authentication, 17 elemental concentrations and 78 ratios of element concentrations were considered (cp. Section 3.3), resulting in a total of 95 variables:

Al, B, Ba, Ca, Co, Cu, Fe, Ga, Mg, Mn, Mo, Ni, Rb, Sr, Te, Tl, Zn, Rb/B, Sr/B, Mo/B, Ba/B, Co/B, Ni/B, Sr/Rb, Mo/Rb, Ba/Rb, Co/Rb, Ni/Rb, Mo/Sr, Ba/Sr, Co/Sr, Ni/Sr, Co/Mo, Mo/Ba, Co/Ba, B/Mg, Rb/Mg, Sr/Mg, Mo/Mg, Ba/Mg, Ca/Mg, Mn/Mg, Fe/Mg, Co/Mg, Ni/Mg, Cu/Mg, Zn/Mg, B/Ca, Rb/Ca, Sr/Ca, Mo/Ca, Ba/Ca, Mn/Ca, Fe/Ca, Co/Ca, Ni/Ca, Cu/Ca, Zn/Ca, B/Mn, Rb/Mn, Sr/Mn, Mo/Mn, Ba/Mn, Fe/Mn, Co/Mn, Ni/Mn, Cu/Mn, Zn/Mn, B/Fe, Rb/Fe, Sr/Fe, Mo/Fe, Ba/Fe, Co/Fe, Ni/Fe, Cu/Fe, Zn/Fe, Mo/Ni, Ba/Ni, Co/Ni, Cu/B, Rb/Cu, Sr/Cu, Mo/Cu, Ba/Cu, Co/Cu, Ni/Cu, B/Zn, Rb/Zn, Sr/Zn, Mo/Zn, Ba/Zn, Co/Zn, Ni/Zn, and Cu/Zn.

## 3. Results and Discussion

### 3.1. Explanation for the Usage of Walnut Kernels

Theoretically, both walnut shell and walnut kernel could be usable for an authentication study. It is reasonable to assume that both parts reflect the elemental pattern of the soil and, thus, the origin.

However, walnuts are mostly traded as shelled goods: in 2018, the percentages of shelled walnuts imported to Europe and Germany were 62% and 81%, respectively [40]. An analytical method developed solely based on the shell would only be applicable to 38% or 19% of potential food fraud, respectively. Additionally, as stated earlier, walnut kernels grow inside the shell and are therefore virtually unaffected by perturbations such as anthropogenic aerosols and soil dust [20]. For these reasons, we decided to use the walnut kernels, as is also practiced in other studies [15,16,17]. Whenever mentioning walnuts samples henceforth, we are referring to walnut kernels.

### 3.2. Selection of Variables for the Chemometric Analysis of 237 Walnut Kernel Samples

From the 47 elements acquired, not all elements were considered for chemometric analysis. Concentrations below the LOQ were obtained for some elements: if the content of those samples exceeded 20%, the respective element was not used for evaluation (this was the case for Ag (22%), Pr (25%), Sm (27%), Dy (32%), Na (36%), Y (42%), Er (53%), Yb (56%), Pb (64%), As (73%), V (75%), Th (78%), Se (78%), Eu (81%), U (85%), Tb (89%), Ho (89%), Cd (91%), Bi (95%), Sc (97%), Li (99%), Tm (99%), Lu (99%), and Be (100%)). Otherwise, the concentration was set to the LOQ level instead of zero, ensuring logarithmic functions to be applicable. For K, the concentrations were over the calibration range for all of the samples; thus, K concentrations were not used for chemometric modeling.

The long-term stability (reproducibility) evaluated by the QC sample deviated between 5.2% (Co) and 27% (Al) (median: 9.2%), except for Te, Tl, Gd, Nd, Ce, and La (54–107%), which were at very low concentrations in the chosen QC sample.

**Figure 2:**
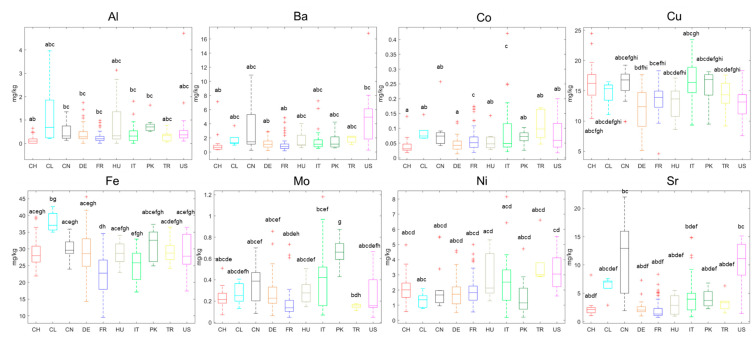
Boxplots for the highly significant elements for the walnuts’ origins after one-way ANOVA testing. Different small letters indicate significant inter-class differences, as determined by Bonferroni post-hoc tests. The “+”-symbol indicates outliers as calculated by Matlab’s boxplot-function (cf. Section 2.5). Data are expressed as mg/kg in walnut lyophilizate.

One-way ANOVA tests indicated that Al, Ba, Co, Cu, Fe, Mo, Ni, and Sr were highly significant (99% confidence level) for the walnut origins. The corresponding boxplots are shown in Figure 2. B, Ga, Mg, Mn, Rb, Te, Tl, and Zn were significant (95% confidence level). Ce, Cr, Gd, La, and Nd, however, showed no significance and were not used for chemometric modeling, also because of the high deviations for the QC sample.

One can observe an increasing tendency where elemental concentration ratios, especially the rare earth elements (REE) among these, are considered for chemometric evaluation besides absolute concentrations [13,14,41,42]. In this way, the model can become more robust [41]. We recently evaluated the benefit of considering concentration ratios in addition to elemental concentrations. Furthermore, we see the possibility of foregoing a drying step for the samples; the water content is no longer important if only elemental ratios are used for chemometric modeling [13]. The concentrations of B, Ba, Ca, Co, Cu, Fe, Mg, Mn, Mo, Ni, Rb, Sr, and Zn were >LOQ for all 237 samples, and for these 13 elements the concentration ratios were calculated. In order to reduce redundant data, duplicate ratios with an interchangeable nominator and denominator were rejected, resulting in 78 element ratios (see Section 2.5).

### 3.3. Chemometric Analysis of the Walnut Samples

#### 3.3.1. Data Investigation and Visualization

**Figure 3:**
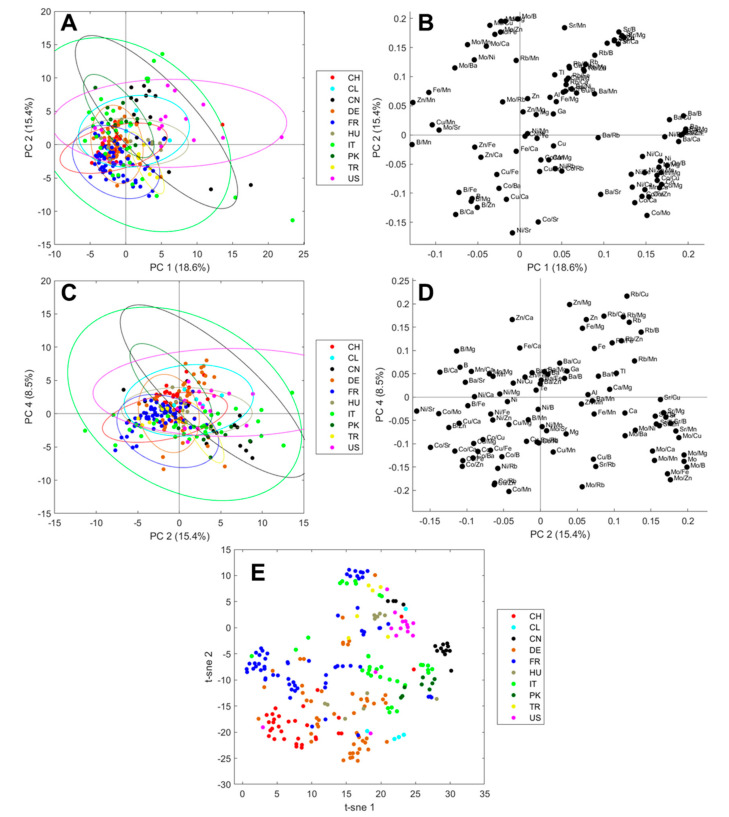
Unsupervised visualization of the multielement data of 237 walnut samples after mean centering and standard deviation scaling using PCA and *t*-SNE. Scores are colored by the origin in the PC1 vs. PC2 plane (**A**) with the corresponding loadings plot (**B**), and the PC2 vs. PC4 plane (**C**) with the corresponding loadings plot (**D**). 95% confidence ellipses were added to the scores plots in (**A**) and (**C**). *t*-SNE plot colored by the origin (**E**).

PCA plots after centering (mean) and scaling (standard deviation) are shown in Figure 3. In the principal component 1 (PC1) vs. PC2 plane, samples from the USA tend to have higher PC1 values and differ most strongly compared to the rest of the samples. In the PC2 vs. PC4 plane, a better visual differentiation can be achieved: Chinese and Pakistani samples are located in the lower right of the scores plot. Swiss and German samples are located in the upper half, while Italian samples tend to have lower PC4 values and French samples tend to have lower PC2 values.

The usage of PCA models for data investigation and visualization is very common for authentication studies; however, it is not always the best choice for visualizing big data sets. The non-linear iterative partial least squares (NIPALS) algorithm focuses on the largest differences in the data set and sets the axis towards the greatest variance [25,43]. Few samples of a minor sample population may get lost in the shuffle [44]. Therefore, we chose *t*-SNE as an additional approach to visualize the data. This can be described as a complementary method, since it focuses on the similarities between two data points rather than the differences [26]. Like PCA, it is an unsupervised model, and the closer two data points the more similar they are. As seen from Figure 3E, the samples tend to cluster according to their origins. In this plot, the clusters seem to be more distinguishable for all sample populations, though the clusters still do overlap and supervised models are needed to determine the origin. However, the boxplots in Figure 2 have already proven that the differentiation of origin is possible; the eight elements presented here show a visual distinction, and for some countries of origin a marker element can already be identified visually. Most apparent is that walnut samples from China and USA contain higher concentrations of Ba and Sr compared with the other walnut samples. The Chinese and US-American samples can be distinguished by Cu, with it being increased for Chinese samples compared to the US ones. Pakistani samples have a higher Mo content. Walnut samples from Chile contain more Fe, and, like Hungarian walnuts, contain more Al.

#### 3.3.2. Influence of the Harvest Year

The fact that the year of harvest has no significant influence on the elemental pattern is recognized in many isotopolomics studies and is considered an advantage of ICP-MS analysis [12,45]. This also applies for walnuts, as examined in a previous study, where the harvesting year and the climatic conditions showed no significant influence on the element pattern [3]. On the other hand, when analyzing the metabolome/proteome the harvest year affected the fatty acid saturation degree and the protein amount [18]. Even for stable isotope analysis, annual differences in the *δ*^2^H-values occur [17]. However, to verify the potential influence of the harvest year for our own data set, French, German, and Italian samples were examined for their potential influence, since most of the walnut samples originated from these three countries and were evenly distributed for three harvest years (see Figure 1). ANOVA tests were calculated, and the highest *F*-value (2.92) was found for Mn for French walnuts; however, this value was smaller than the critical *F*-value of 3.15 (0.05 significance level). Thus, none of the 17 elements showed a significant influence with regard to the harvest years.

Additionally, the PCA score plot of all ten origins (Figure 3A) was colored by the respective harvest years and is shown in Figure S1A (Supplementary Materials). For a better visual comparison, both score plots are shown in Figure S1B for direct comparison. While the scores tend to cluster according to their origins, as discussed above, no clustering according to the harvest years is noticeable. Consequently, the origin has a higher influence on the data than the harvest year. This might enable this study to be suitable for the prediction of new samples without the necessity of new reference samples in future years. In fact, this is a further advantage of this method, since the origin of walnut samples can be predicted at the beginning of the next harvest season without any need for new reference samples.

#### 3.3.3. Influence of the Cultivar

Besides the harvest year, the cultivar—in other words, the genotype—may have an influence on the walnut’s isotopolome and may cause an unwanted bias in this origin authentication study. In previous studies, a dependency of element uptake for different walnut cultivars was found for Cu, K, Fe, Mn, and Zn [3,46]. However, these studies mainly concerned a physiological-nutritional analysis of walnuts, and the number of samples was relatively small, with 24 and 9, respectively. Considering K, its potential influence can be considered as irrelevant anyway, since the signal intensity of K, as the element with the highest concentration in the walnut kernel, was above the calibration range and, therefore, K was not taken into account for statistical evaluation (see Section 3.2).

Another study implies that the genotype solely has only a secondary effect on the isotopolome: Juranović Cindrić et al. analyzed the elemental composition of *Juglans nigra* walnut samples, which are another species compared to *Juglans regia*, as investigated in this study. The authors compared the elemental concentrations to the literature values of *Juglans regia* and found similar results [21]. Thus, when the elemental concentrations of different *Juglans* species are similar, the elemental concentrations of different cultivars, a taxonomic rank below the species, should be similar as well.

It should be emphasized that the potential influence of the cultivar would be problematic for the origin authentication when all samples from one country consisted of a cultivar which would reversely originate solely from this country. This is not the case for the present data set as presented in Table S2. Considering two major cultivars, for example, 47 walnut “Lara” samples originate from Switzerland (2), Germany (2), France (30), and Italy (13). Twenty walnut “Chandler” samples originate from Switzerland (2), Chile (1), China (2), Italy (11), Turkey (1), and the USA (3) (number in brackets indicates the sample size per origin). Consequently, no origin or cultivar was overrepresented. Additionally, PCA plots were calculated for 120 walnut samples, for which the information of the cultivar was available and there were at least three samples of the respective cultivar. The scores plots are shown in Figure S2 (Supplementary Materials), and the scores are colored by origin and cultivar for direct comparison. As seen from Figure S2, the samples do not cluster with respect to the cultivar; thus, the cultivar has a secondary effect on the isotopolome.

#### 3.3.4. Classification of the Geographical Origin

For the origin differentiation, 237 samples from ten origins were considered. Mean concentrations with standard deviations are given in Table S6 (supporting information). For all combinations stated in Table 1, a model was calculated to find the best suited settings. As the response variable, the overall accuracy was investigated, and the overall accuracies are stated in Tables S7 and S8 (Supplementary Materials). As seen from the results, the choice of the classification method has a major impact on the model’s performance compared to the data pre-treatment. Especially for the different center and scale approaches, the accuracies only differ in the second decimal. Using stratified nested cross validation, the best accuracy of 72.9% ± 1.6% was found after center (mean) and scale (standard deviation) using SVM. For leave-one-out cross validation, the best accuracy was reached at an improved level of 75.5%, also achieved after center (mean), scale (standard deviation), and SVM. The corresponding confusion matrices are shown in Table 2 and Table 3, respectively. To compare these two models, both classification models were evaluated by calculating the sensitivity and the specificity per class to examine the type I and type II errors [47,48]. For the stratified nested cross validation sensitivity, the scores ranged from 20.0% to 84.6%, and the specificity ranged from 25.5% to 87.8%. Meanwhile, for the leave-one-out cross validation, the sensitivity ranged from 16.7% to 86.7%, and the specificity ranged from 20.0% to 100%. The Turkish walnuts are the blind spot for both classification models, with comparably low sensitivity scores of 20.0% (stratified nested cross validation) and 16.7% (leave-one-out cross validation), respectively. In the future, the prediction accuracy of Turkish walnuts may be improved by data fusion—i.e., combining our data set with other omics-disciplines or isotope ratio analysis [9,49]. When comparing the sensitivities per class, the values of the nested cross validation do show less variation or, in other words, the standard deviation is lower. Particularly for the two origins with the fewest number of samples (Chile and Turkey), the accuracies are superior by 3 and 6 percentage points. The stratified approach (see Section 2.5) used for the calculation of the test and training of the nested cross validation set may positively influence the sample’s distribution and lead to more evenly distributed accuracies. Therefore, we prefer the classification model validated by stratified nested cross validation, despite the slightly reduced accuracy of 2.7 percentage points. To our knowledge, in the literature authors only seldom comment on their choice of which validation method to apply [15,47]. Although it is mentioned that, via stratified nested cross validation, a generally valid model can be calculated which does not lack overfitting, while leave-one-out cross validation is more prone to this issue [12,38,39,50], we would like to encourage the reader to apply both validation strategies and compare the results.

The current sample preparation includes a drying step that is both time and energy consuming and could be optimized for environmental reasons [51]. Without a drying step, this method would be applicable in the food industry where the incoming goods inspection should be carried out as fast as possible. Without drying though, the element contents cannot be expressed in relation to the dry matter, which makes it difficult to evaluate the walnut samples chemometrically. When considering only elemental concentration ratios, however, a comparison is possible. Therefore, the calculation of the classification model (SVM, stratified nested cross validation) was repeated using only the 78 elemental ratios listed in Section 2.5. The prediction accuracy dropped as expected, but only marginally: an overall accuracy of 72.2% ± 1.6% was achieved using stratified nested cross validation (before: 72.9% ± 1.6%). For the sake of completeness, leave-one-out cross validation led to 74.7% (before: 75.5%). The respective loss of accuracy is not significant, and, in this way, fresh walnut samples can also be analyzed in the future without an obligatory drying step

#### 3.3.5. Classification of the Regional Origin of French, German and Italian Walnuts

For France, Germany, and Italy, more than 30 samples could be acquired and, thanks to our project partners, highly authentic samples with detailed and reliable information regarding the origin on a regional level were available (see Table S2). Therefore, the potential of the analytical method to predict the origin even on a regional level was investigated. Mean concentrations with standard deviations for the regions are given in Tables S9–S11 (Supporting Information).

For France, the data set existed of 53 samples from the four regions Auvergne-Rhône-Alpes (ten samples), Nouvelle-Aquitaine (26 samples), Occitanie (11 samples), and Pays de la Loire (six samples). A PCA was calculated for these samples, and clusters can already be recognized for the origin, as seen in the scores and loadings plot shown in Figure S3A,B (Supplementary Materials), respectively. Classification models were calculated for this issue with all combinations stated in Table 1, except leave-one-out cross validation, because of the results in the previous section. The best accuracy of 91.4% ± 2.1% was found after a log_10_ transformation and an SVM classification model (Table S12, supporting information). The corresponding confusion matrix is shown in Table 4. The separation of samples from Pays de la Loire succeeded almost without error with a sensitivity of 99%. To identify the elements causing the separation, an ANOVA test was calculated. The boxplots of the elements with the highest F-values are shown in Figure S4A (Supplementary Materials). As can be seen here, the Pays de la Loire can be well distinguished from any other French region because of the significant higher concentrations of Co. Ba and Sr are also important for the regional differentiation.

Not yet considered was the Noix de Grenoble, the only walnut with a geographical indication (in French: appellation d’origine protégée (AOP)). These are walnuts originating from certain municipalities in the départments of Isère, Drôme, and Savoie [52]. The three départements are located in Auvergne-Rhône-Alpes; thus, it would be rather challenging to conduct a sub-regional authentication study. However, the promising results obtained so far give the possibility to follow up this classification issue in the future.

For Germany and Italy, the data set was analogously analyzed: PCA scores plots are shown in Figure S3, and the confusion matrices are shown in Table 5 and Table 6, respectively.

The German data set existed of 48 samples from the four German federal states Baden-Württemberg (9 samples), Hesse (16 samples), Lower Saxony (14 samples), and North Rhine-Westphalia (9 samples). The best accuracy of 77.4% ± 2.5% was achieved after center (median) and scale (standard deviation) with an SVM model (Table S13, Supporting Information). Compared to the French samples, the prediction is not as good; here, especially, the samples from Lower Saxony are likely to be misclassified. Again, ANOVA was applied to identify the elements showing significant differences, and the boxplots of the three elements with the highest *F*-values are shown in Figure S4B. The calculated classification model tends to confuse walnut samples from North Rhine-Westphalia and Lower Saxony, and this is also observable in the boxplots showing similar distributions for Fe and Rb for these regions. Cu shows significant differences between the four regions, but the distributions overlap, making the differentiation difficult.

From Italy, 32 samples could be acquired from Campania, Napoli (four samples); Piedmont, Cuneo (13 samples); Veneto, Padova (five samples); and Veneto, Rovigo (ten samples). An overall accuracy of 94.2% ± 2.8% can be achieved after log_10_ transformation with an SVM model (Table S14, supporting information). The three Italian regions examined are geographically separated, which may explain the good predictive power, but even within the Veneto region, accurate classification is possible. Most importantly, Fe, Sr, and Zn are relevant marker elements for the differentiation as outlined by an ANOVA test and shown in the corresponding boxplots in Figure S4C.

It should be pointed out that an entirely unknown walnut sample would have to be predicted by the multiclass model first before applying the regional models presented in this section. Mathematically, when applying the classification models one after the other, the prediction accuracies would have to be multiplied (e.g., the chance that a walnut sample will be correctly be predicted as Italian and originating from Napoli would be 0.685 · 0.975 ≡ 66.8%). Furthermore, it should be noted that, due to the comparably lower number of samples for the regional classification models, a potential over-fitting is more likely to occur. Thus, more reference samples should be acquired and measured in the future to confirm and enhance the model’s reliability.

#### 3.3.6. Further Evaluation of the Classification Models’ Performance

Classification models are usually evaluated based on their accuracy, sensitivity, and specificity [47,48,50]. Especially the accuracy is the most important parameter, and of course higher accuracies are desirable. At the same time, the sole expressive power of the accuracy should not be exaggerated. Regarding the classification models of this study, the model for all origins reached 73%, and that for the regional levels in Italy 94%. Both classification issues were validated using nested cross validation; hence, the potential risk of overfitting could be reduced [38,39].

At first glance, 94% seems to be better than 73%, and this appears to be almost paradoxical when considering that the differentiation on a regional level is better than on a worldwide level, although the geographical distances shrunk and, thus, the soil should be more similar. However, it is unreasonable to make a statement about which model is better, because the models are hardly possible to compare with each other—they deal with different issues, and the input data and the classification models’ sizes are different.

To our knowledge, there is no additional parameter to evaluate the model’s performance and allow us to compare different classification models. Hence, we examined a quotient similar to the signal-to-noise ratio. This parameter is commonly used in analytical chemistry to evaluate a measurement signal. The sole signal’s intensity does not allow a meaningful assessment of the signal’s quality; instead, the noise has to be considered as well. Then, the higher the signal-to-noise ratio, the more reliable the measurements, and the more robust the results. Regarding the classification models, the signal corresponds to the model’s accuracy. The noise is the theoretical accuracy when distributing the samples at random. Mathematically, this value equates to the reciprocal number of classes. Hence, this value correlates to the model’s size, and since models with more classes are more challenging to calculate, it accounts for the classification model’s difficulty. With the accuracy-to-random ratio, the classification models can be further evaluated:

In Table 7, the characteristics of the classification models discussed in Section 3.3.4 and Section 3.3.5 are stated. For comparison, four additional binary classification models were calculated and included in this table: on the one hand, three issues targeting the distinction between Germany and an exporting country (Chile, China, and the USA, respectively); on the other hand, Europe (combining Switzerland, France, Germany, Hungary, and Italy) vs. not-Europe (combining Chile, China, Pakistan, Turkey, and the USA). The very high percentages (>95%) for these binary models should be noted; however, it is maximally possible to be twice as good compared to a random distribution. On the contrary, the classification models on the regional level outperform the random distribution by three times, and the worldwide classification model outperforms the random distribution even by seven, emphasizing the high performance of this model. For the sake of completeness, we also calculated all binary 1‑vs.‑1 classification models—i.e., all possible two-paired combinations of the ten countries of origins. The results are given in Table S15 (Supplementary Materials), and the accuracies range from 81.7% to 99.0%. Now, also Hungarian and Turkish samples reach fairly good accuracies (>80%), emphasizing again that the calculated accuracies always have to be set in relation to the complexity of the model—i.e., the number of classes. The sole expressive power of the accuracy has limited information value.

It should therefore be pointed out that the model’s accuracy possesses a limited expressive power. However, this is not a generic criticism of the usage of binary classification models; such binary issues often match the authentication problems in practice—e.g., the differentiation of the most expensive white truffle from its cheaper counterfeit [53], or the distinction of a regional product with a protected geographical indication from foreign samples [42].

For walnuts, however, we do not see any options to simplify the multiclass models to binary models, since global trade and/or import to Europe is strongly interlinked, and therefore only the multiclass approach is reasonable for worldwide differentiation.

## 4. Conclusions

The elemental analysis of walnut with ICP-MS in combination with chemometrics proved to be a powerful technique for geographical origin differentiation on a worldwide and regional level. Although the REE were not considered to be due to too-low concentrations, the worldwide origin was successfully predicted with an overall accuracy of 73%. The most important variables were Al, Ba, Co, Cu, Fe, Mo, Ni, and Sr. No significant loss of accuracy was observed when only the elemental ratios were considered, so fresh walnut samples can be analyzed without the need for a drying step. On a regional level in France, Germany, and Italy, the differentiation of walnut samples was possible, with overall accuracies of 91%, 77%, and 94%, respectively. In the future, we want to broaden walnut authentication with the Noix de Grenoble. Harvest year and cultivar showed no observable influence, which makes this method suitable for predicting new samples without the need for reference samples in future years.

## Figures and Tables

**Table 1:** Overview of the settings for data pre-treatment, classification methods with hyperparameters, and validation for the calculation of the classification models.

Data Pre-Treatment	Classification Method	Validation
(i) no pre-treatment	(1) linear discriminant analysis, LDA γ = 0	(a) stratified nested cross validation
(ii) log10	(2) support vector machine, SVM polynomial order = 2 box constraint level = 1 coding: one vs. one	(b) leave-one-out cross validation
(iii) center (mean) and scale (standard deviation)	(3) subspace discriminant, SSD number learning cycles = 30	
(iv) center (median) and scale (standard deviation)	(4) random forest, RF split criterion: Gini’s diversity index max. number of splits = 100 min leaf size = 1 surrogate: off	
(v) center (median) and scale (range)		
(vi) center (median) and scale (interquartile range)		

**Table 2:** Confusion matrix for the classification of all 237 walnut samples with the SVM model, resulting in a 72.9% ± 1.6% overall accuracy using stratified nested cross validation. The mean values of 20 predictions are given.

		Predicted Origin										
		Switzerland	Chile	China	Germany	France	Hungary	Italy	Pakistan	Turkey	United States	Sensitivity [%]
Actual Origin	Switzerland	22.1	0.0	0.0	6.4	1.6	0.0	0.0	0.0	0.0	1.0	71.3
	Chile	0.0	3.3	0.7	1.0	0.0	0.0	0.0	0.0	0.0	0.0	66.0
	China	0.7	0.0	12.7	1.0	0.4	0.0	0.0	0.0	0.3	0.0	84.3
	Germany	2.8	0.2	0.0	39.5	6.3	0.5	0.1	0.0	0.1	0.6	79.0
	France	1.2	0.0	0.0	6.8	53.3	0.8	0.5	0.0	0.5	0.1	84.6
	Hungary	0.0	0.0	0.0	4.9	0.2	4.8	0.0	1.0	0.1	0.0	43.6
	Italy	0.0	0.9	0.1	2.6	4.7	0.3	22.6	0.7	1.0	0.4	68.5
	Pakistan	0.0	0.0	0.4	2.0	0.0	1.1	0.3	4.4	0.0	0.0	54.4
	Turkey	0.7	0.0	0.0	1.3	1.5	0.4	1.1	0.0	1.2	0.0	20.0
	United States	1.0	0.3	0.7	1.0	0.0	0.1	1.2	0.3	1.6	8.9	59.3
	specificity [%]	77.8	72.5	87.5	59.6	78.6	61.1	87.8	69.0	25.5	81.7	

**Table 3:** Confusion matrix for the classification of all 237 walnut samples with the SVM model, resulting in a 75.5% overall accuracy using leave-one-out cross validation.

		Predicted Origin										
		Switzerland	Chile	China	Germany	France	Hungary	Italy	Pakistan	Turkey	United States	Sensitivity [%]
Actual Origin	Switzerland	24	0	0	5	1	0	0	0	0	1	77.4
	Chile	0	3	1	1	0	0	0	0	0	0	60.0
	China	1	0	13	1	0	0	0	0	0	0	86.7
	Germany	2	0	0	41	6	0	0	0	0	1	82.0
	France	1	0	0	6	54	1	0	0	1	0	85.7
	Hungary	0	0	0	5	0	5	0	1	0	0	45.5
	Italy	0	0	0	2	4	1	24	1	1	0	72.7
	Pakistan	0	0	0	2	0	1	0	5	0	0	62.5
	Turkey	0	0	0	2	1	0	2	0	1	0	16.7
	United States	1	0	1	1	0	0	1	0	2	9	60.0
	specificity [%]	82.8	100.0	86.7	62.1	81.8	62.5	88.9	71.4	20.0	81.8	

**Table 4:** Confusion matrix for the classification of 53 French walnut samples with the SVM model, resulting in a 91.4% ± 2.1% overall accuracy using stratified nested cross validation. The mean values of 20 predictions are given.

		Predicted Regional Origin				
		Pays de la Loire	Nouvelle-Aquitaine	Auvergne-Rhône-Alpes	Occitanie	Sensitivity [%]
Actual Regional Origin	Pays de la Loire	6.0	0.1	0.0	0.0	99.2
	Nouvelle-Aquitaine	0.0	25.2	0.0	0.9	96.7
	Auvergne-Rhône-Alpes	0.0	1.3	8.5	0.2	85.0
	Occitanie	0.3	1.2	0.8	8.9	80.5
	specificity [%]	96.0	91.0	91.9	89.4	

**Table 5:** Confusion matrix for the classification of 48 German walnut samples with the SVM model, resulting in a 77.4% ± 2.5% overall accuracy using stratified nested cross validation. The mean values of 20 predictions are given.

		Predicted Regional Origin				
		North Rhine-Westphalia	Baden-Württemberg	Hesse	Lower Saxony	Sensitivity [%]
Actual Regional Origin	North Rhine-Westphalia	6.3	0.0	0.0	2.7	70.0
	Baden-Württemberg	0.0	15.1	0.1	0.9	94.1
	Hesse	0.0	2.0	12.0	0.0	85.7
	Lower Saxony	2.2	2.6	0.4	3.8	42.2
	specificity [%]	74.1	76.6	96.4	51.4	

**Table 6:** Confusion matrix for the classification of 32 Italian walnut samples with the SVM model, resulting in a 94.2% ± 2.8% overall accuracy using stratified nested cross validation. The mean values of 20 predictions are given.

		Predicted Regional Origin				
		Veneto, Padova	Piedmont, Cuneo	Veneto, Rovigo	Campania, Napoli	Sensitivity [%]
Actual Regional Origin	Veneto, Padova	5.0	0.0	0.0	0.0	100.0
	Piedmont, Cuneo	0.0	11.7	1.3	0.1	89.6
	Veneto, Rovigo	0.0	0.4	9.6	0.0	96.0
	Campania, Napoli	0.0	0.0	0.1	3.9	97.5
	specificity [%]	100.0	96.7	87.3	98.7	

**Table 7:** Evaluation of different classification models for the authentication of walnuts.

Parameter		Accuracy [%]	Number of Classes	Random Distribution [%]	Accuracy-to-Random Ratio
Variables and Equations		*a*	*n*	*r =* 100%*/n*	*a/r*
global differentiation	all countries of origin	72.9	10	10	7.29
regional differentiations	FR	91.4	4	25	3.66
	DE	77.4	4	25	3.10
	IT	94.2	4	25	3.77
binary classification models	CN vs. DE	99.5	2	50	1.99
	US vs. DE	96.6	2	50	1.93
	CL vs. DE	98.2	2	50	1.96
	Europe vs. not-Europe	92.4	2	50	1.85

## Supplementary Materials

The following figures and tables are available online at https://www.mdpi.com/2304-8158/9/11/1708/s1: Figure S1: PCA plots for the comparison on the influence of harvest year vs. origin; Figure S2: PCA plots for the comparison on the influence of cultivar vs. origin; Figure S3: PCA plots for the regional differentiation of walnut samples within France, Germany and Italy; Figure S4: Boxplots for the significant elements for the walnuts’ origins authentication on a regional level in France, Germany and Italy after one-way ANOVA testing. Data expressed as mg/kg in walnut lyophilizate; Table S1: Reagents and materials used in this study; Table S2: Detailed information of all walnut samples analyzed in this study with cultivar, origin and harvest year; Table S3: Microwave digestion procedure; Table S4: Limit of detection (LOD) and limit of quantitation (LOQ) for the measured isotopes for the HR-ICP-MS instrument. Additionally, the respective internal standard element is given; Table S5: Instrumental conditions and measurement parameters for HR-ICP-MS; Table S6: Mean elemental concentrations for the walnut countries of origin in mg/kg; Table S7: Overall accuracy with standard deviation for different data pre-treatment and classification methods for the predictions of all walnut samples using stratified nested cross validation; Table S8: Overall accuracy with standard deviation for different data pre-treatment and classification methods for the predictions of all walnut samples using leave-one-out cross validation; Tables S9–S11: Mean elemental concentrations for the French, German, and Italian walnut regions; Tables S12–S14: Overall accuracy with standard deviation for different data pre-treatment and classification methods for the French, German, and Italian walnut samples using stratified nested cross validation; Table S15: Overall accuracies of binary classification using stratified nested cross-validation of 20 repetitions (classification method: quadratic SVM; data pre-treatment: log10 transformation).

## References

[1] Martínez M.L., Labuckas D.O., Lamarque A.L., Maestri D.M. (2010). Walnut (*Juglans regia* L.): Genetic resources, chemistry, by-products. J. Sci. Food Agric..

[2] Janick J., Paull R.E. (2008). The Encyclopedia of Fruit and Nuts.

[3] Momchilova S., Arpadjan S., Blagoeva E. (2016). Accumulation of microelements Cd, Cu, Fe, Mn, Pb, Zn in walnuts (*Juglans regia* L.) depending on the cultivar and the harvesting year. Bulg. Chem. Commun..

[4] Creydt M., Fischer M. (2018). Omics approaches for food authentication. Electrophoresis.

[5] McGrath T.F., Shannon M., Chevallier O.P., Ch R., Xu F., Kong F., Peng H., Teye E., Akaba S., Wu D. (2020). Food Fingerprinting: Using a two-tiered approach to monitor and mitigate food fraud in rice. J. AOAC Int..

[6] Schelm S., Siemt M., Pfeiffer J., Lang C., Tichy H.-V., Fischer M. (2020). Food Authentication: Identification and Quantitation of Different Tuber Species via Capillary Gel Electrophoresis and Real-Time PCR. Foods.

[7] Mannino G., Gentile C., Maffei M.E. (2019). Chemical partitioning and DNA fingerprinting of some pistachio (*Pistacia vera* L.) varieties of different geographical origin. Phytochemistry.

[8] Grazina L., Amaral J.S., Mafra I. (2020). Botanical origin authentication of dietary supplements by DNA-based approaches. Compr. Rev. Food Sci. Food Saf..

[9] Kelly S., Heaton K., Hoogewerff J. (2005). Tracing the geographical origin of food: The application of multi-element and multi-isotope analysis. Trends Food Sci. Technol..

[10] Aceto M. (2016). The use of ICP-MS in food traceability. Advances in Food Traceability Techniques and Technologies.

[11] Drivelos S.A., Georgiou C.A. (2012). Multi-element and multi-isotope-ratio analysis to determine the geographical origin of foods in the European Union. TrAC Trends Anal. Chem..

[12] Richter B., Gurk S., Wagner D., Bockmayr M., Fischer M. (2019). Food authentication: Multi-elemental analysis of white asparagus for provenance discrimination. Food Chem..

[13] Segelke T., von Wuthenau K., Neitzke G., Mueller M.-S., Fischer M. (2020). Food Authentication: Species and origin determination of truffles (*Tuber* spp.) by inductively coupled plasma mass spectrometry (ICP-MS) and chemometrics. J. Agric. Food Chem..

[14] Oddone M., Aceto M., Baldizzone M., Musso D., Osella D. (2009). Authentication and traceability study of hazelnuts from Piedmont, Italy. J. Agric. Food Chem..

[15] Esteki M., Farajmand B., Amanifar S., Barkhordari R., Ahadiyan Z., Dashtaki E., Mohammadlou M., Vander Heyden Y. (2017). Classification and authentication of Iranian walnuts according to their geographical origin based on gas chromatographic fatty acid fingerprint analysis using pattern recognition methods. Chemom. Intell. Lab. Syst..

[16] Gu X., Zhang L., Li L., Ma N., Tu K., Song L., Pan L. (2018). Multisource fingerprinting for region identification of walnuts in Xinjiang combined with chemometrics. J. Food Process. Eng..

[17] Krauß S., Vieweg A., Vetter W. (2020). Stable isotope signatures (δ2H-, δ13C-, δ15N-values) of walnuts (*Juglans regia* L.) from different regions in Germany. J. Sci. Food Agric..

[18] Popescu R., Ionete R.E., Botoran O.R., Costinel D., Bucura F., Geana E.I., Alabedallat Y.F.J., Botu M. (2019). 1H-NMR profiling and carbon isotope discrimination as tools for the comparative assessment of walnut (*Juglans regia* L.) cultivars with various geographical and genetic origins—A preliminary study. Molecules.

[19] Valdés A., Beltrán A., Mellinas C., Jiménez A., Garrigós M.C. (2018). Analytical methods combined with multivariate analysis for authentication of animal and vegetable food products with high fat content. Trends Food Sci. Technol..

[20] Rodushkin I., Engström E., Sörlin D., Baxter D. (2008). Levels of inorganic constituents in raw nuts and seeds on the Swedish market. Sci. Total Environ..

[21] Juranović Cindrić I., Zeiner M., Hlebec D. (2018). Mineral composition of elements in walnuts and walnut oils. Int. J. Environ. Res. Public Health.

[22] Moodley R., Kindness A., Jonnalagadda S.B. (2007). Elemental composition and chemical characteristics of five edible nuts (almond, Brazil, pecan, macadamia and walnut) consumed in Southern Africa. J. Environ. Sci. Heal. Part B.

[23] Ozyigit I.I., Uras M.E., Yalcin I.E., Severoglu Z., Demir G., Borkoev B., Salieva K., Yucel S., Erturk U., Solak A.O. (2018). Heavy Metal Levels and Mineral Nutrient Status of Natural Walnut (*Juglans regia* L.) Populations in Kyrgyzstan: Nutritional Values of Kernels. Biol. Trace Elem. Res..

[24] Food and Agriculture Organization of the United Nations (FAOSTAT) Values of Agricultural Production and Trade of Walnuts in shell. http://www.fao.org/faostat/en/#home.

[25] Wold S., Esbensen K., Geladi P. (1987). Principal component analysis. Chemom. Intell. Lab. Syst..

[26] Maaten L.V.D., Hinton G. (2008). Visualizing data using t-SNE. J. Mach. Learn. Res..

[27] Zhao H., Zhang S., Zhang Z. (2017). Relationship between multi-element composition in tea leaves and in provenance soils for geographical traceability. Food Control.

[28] Cubero-Leon E., Peñalver R., Maquet A. (2014). Review on metabolomics for food authentication. Food Res. Int..

[29] Kim H.-Y. (2014). Analysis of variance (ANOVA) comparing means of more than two groups. Restor. Dent. Endod..

[30] Bland J.M., Altman D.G. (1995). Multiple significance tests: The Bonferroni method. Bmj.

[31] The Mathworks Visualize Summary Statistics with Box Plot. https://de.mathworks.com/help/stats/boxplot.html.

[32] Spruyt V. How to Draw a Covariance Error Ellipse?. https://www.visiondummy.com/2014/04/draw-error-ellipse-representing-covariance-matrix/.

[33] Cortes C., Vapnik V. (1995). Support-vector networks. Mach. Learn..

[34] Bachmann R., Klockmann S., Haerdter J., Fischer M., Hackl T. (2018). 1H NMR spectroscopy for determination of the geographical origin of hazelnuts. J. Agric. Food Chem..

[35] The Mathworks Framework for Ensemble Learning. https://de.mathworks.com/help/stats/framework-for-ensemble-learning.html.

[36] Breiman L. (2001). Random forests. Mach. Learn..

[37] Gerretzen J., Szymańska E., Jansen J.J., Bart J., van Manen H.-J., van den Heuvel E.R., Buydens L.M. (2015). Simple and effective way for data preprocessing selection based on design of experiments. Anal. Chem..

[38] Krstajic D., Buturovic L.J., Leahy D.E., Thomas S. (2014). Cross-validation pitfalls when selecting and assessing regression and classification models. J. Chemin..

[39] Varma S., Simon R. (2006). Bias in error estimation when using cross-validation for model selection. BMC Bioinform..

[40] Food and Agriculture Organization of the United Nations (FAOSTAT) Import Quantity of shelled and in-shell Walnuts. http://www.fao.org/faostat/en/#home.

[41] Bitter N.Q., Fernandez D.P., Driscoll A.W., Howa J.D., Ehleringer J.R. (2020). Distinguishing the region-of-origin of roasted coffee beans with trace element ratios. Food Chem..

[42] Zettl D., Bandoniene D., Meisel T., Wegscheider W., Rantitsch G. (2017). Chemometric techniques to protect the traditional Austrian pumpkin seed oil. Eur. J. Lipid Sci. Technol..

[43] Ballabio D., Todeschini R. (2009). Multivariate classification for qualitative analysis. Infrared Spectrosc. Food Qual. Anal. Control.

[44] Mishra P., Nordon A., Tschannerl J., Lian G., Redfern S., Marshall S. (2018). Near-infrared hyperspectral imaging for non-destructive classification of commercial tea products. J. Food Eng..

[45] Drivelos S.A., Danezis G.P., Haroutounian S.A., Georgiou C.A. (2016). Rare earth elements minimal harvest year variation facilitates robust geographical origin discrimination: The case of PDO “Fava Santorinis”. Food Chem..

[46] Cosmulescu S.N., Baciu A., Achim G., Mihai B., Trandafir I. (2009). Mineral composition of fruits in different walnut (*Juglans regia* L.) cultivars. Not. Bot. Horti Agrobot. Cluj-Napoca.

[47] Latorre C.H., Crecente R.P., Martín S.G., García J.B. (2013). A fast chemometric procedure based on NIR data for authentication of honey with protected geographical indication. Food Chem..

[48] da Costa N.L., Ximenez J.P.B., Rodrigues J.L., Barbosa F., Barbosa R. (2020). Characterization of Cabernet Sauvignon wines from California: Determination of origin based on ICP-MS analysis and machine learning techniques. Eur. Food Res. Technol..

[49] Borràs E., Ferré J., Boqué R., Mestres M., Aceña L., Busto O. (2015). Data fusion methodologies for food and beverage authentication and quality assessment—A review. Anal. Chim. Acta.

[50] Arndt M., Rurik M., Drees A., Bigdowski K., Kohlbacher O., Fischer M. (2020). Comparison of different sample preparation techniques for NIR screening and their influence on the geographical origin determination of almonds (*Prunus dulcis* MILL.). Food Control.

[51] Anastas P., Eghbali N. (2010). Green chemistry: Principles and practice. Chem. Soc. Rev..

[52] Comité Interprofessionnel de la Noix de Grenoble Noix de Grenoble, zone de Production. https://www.aoc-noixdegrenoble.com/terroir/zone-de-production/.

[53] Segelke T., Schelm S., Ahlers C., Fischer M. (2020). Food Authentication: Truffle (*Tuber* spp.) Species Differentiation by FT-NIR and Chemometrics. Foods.

