# Supplementary material for: Origin Determination of Walnuts (Juglans regia L.) on a Worldwide and Regional Level by Inductively Coupled Plasma Mass Spectrometry and Chemometrics
Source: Foods. 2020 Nov 20;9(11):1708. doi: 10.3390/foods9111708 (PMC7699883; doi:10.3390/foods9111708)
Supplement: Supplementary file 1 [file foods-09-01708-s001.pdf]

Supplementary Materials

Origin determination of walnuts (*Juglans regia* L.) on a worldwide and regional level by inductively coupled plasma mass spectrometry and chemometrics

Torben Segelke [1,], Kristian von Wuthenau [1,] Anita Kuschnereit [1], Marie-Sophie Müller [1] and Markus Fischer [1,*]

[1] HAMBURG SCHOOL OF FOOD SCIENCE; Institute of Food Chemistry, University of Hamburg, Grindelallee 117, 20146 Hamburg, Germany; torben.segelke@chemie.uni-hamburg.de (T.S.); kristian.wuthenau@chemie.uni-hamburg.de (K.v.W), anita.kuschnereit@studium.uni-hamburg.de (A.K.); marie-sophie.mueller@studium.uni-hamburg.de (M.M), Markus.Fischer@uni-hamburg.de (M.F.)

* Correspondence: Markus.Fischer@uni-hamburg.de; Tel.: +49-40-42838-4357/59

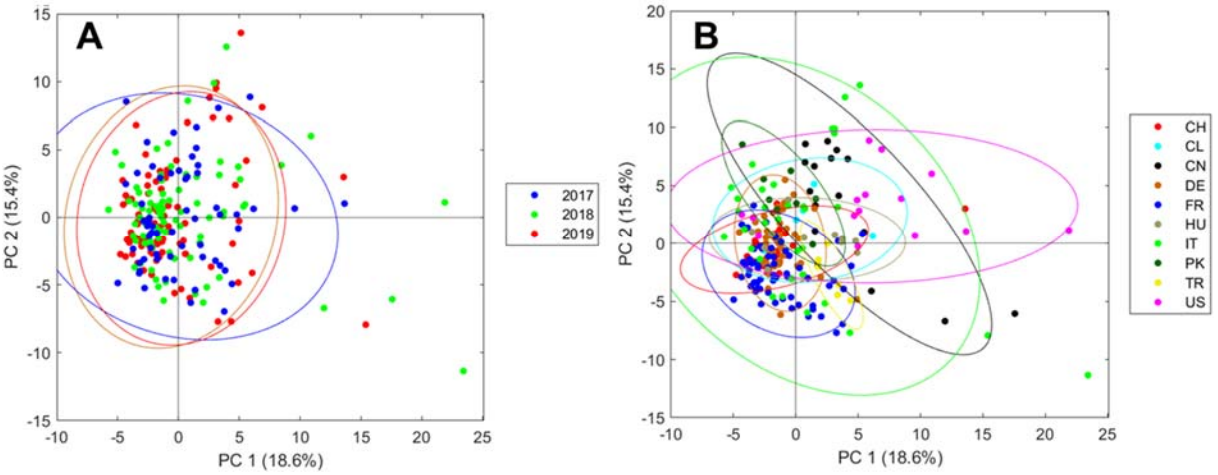

Figure S1: PCA scores plots for the comparison on the influence of harvest year vs. origin, with 95% confidence ellipses. The sample set is colored by the harvest year (A) and the origin (B).

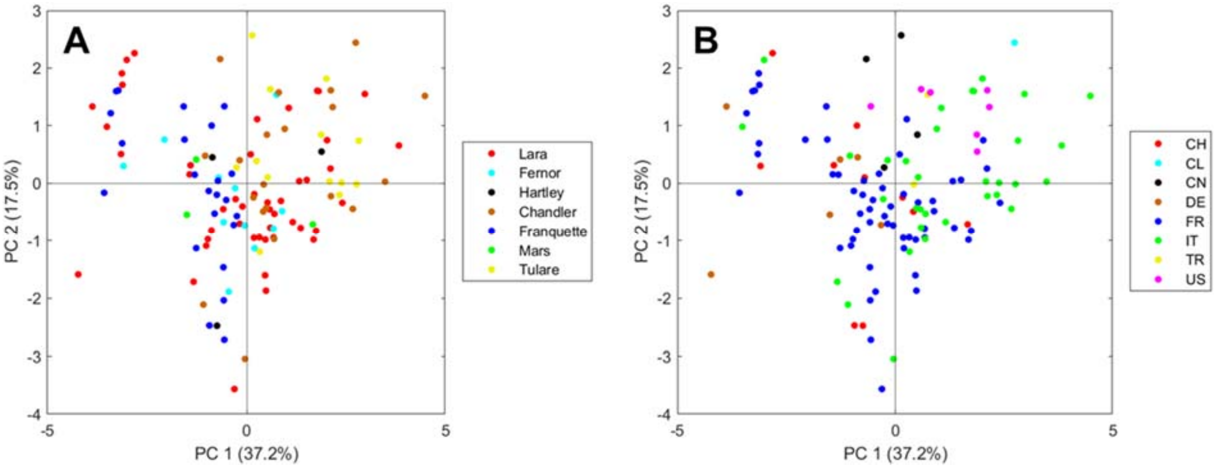

Figure S2: PCA scores plots comparison on the influence of cultivar vs. origin. No 95% confidence ellipses were added because of the small numbers (≤3) of some classes (e.g. Hartley, Mars, Chile). The sample set is colored by the cultivar (A) and the origin (B).

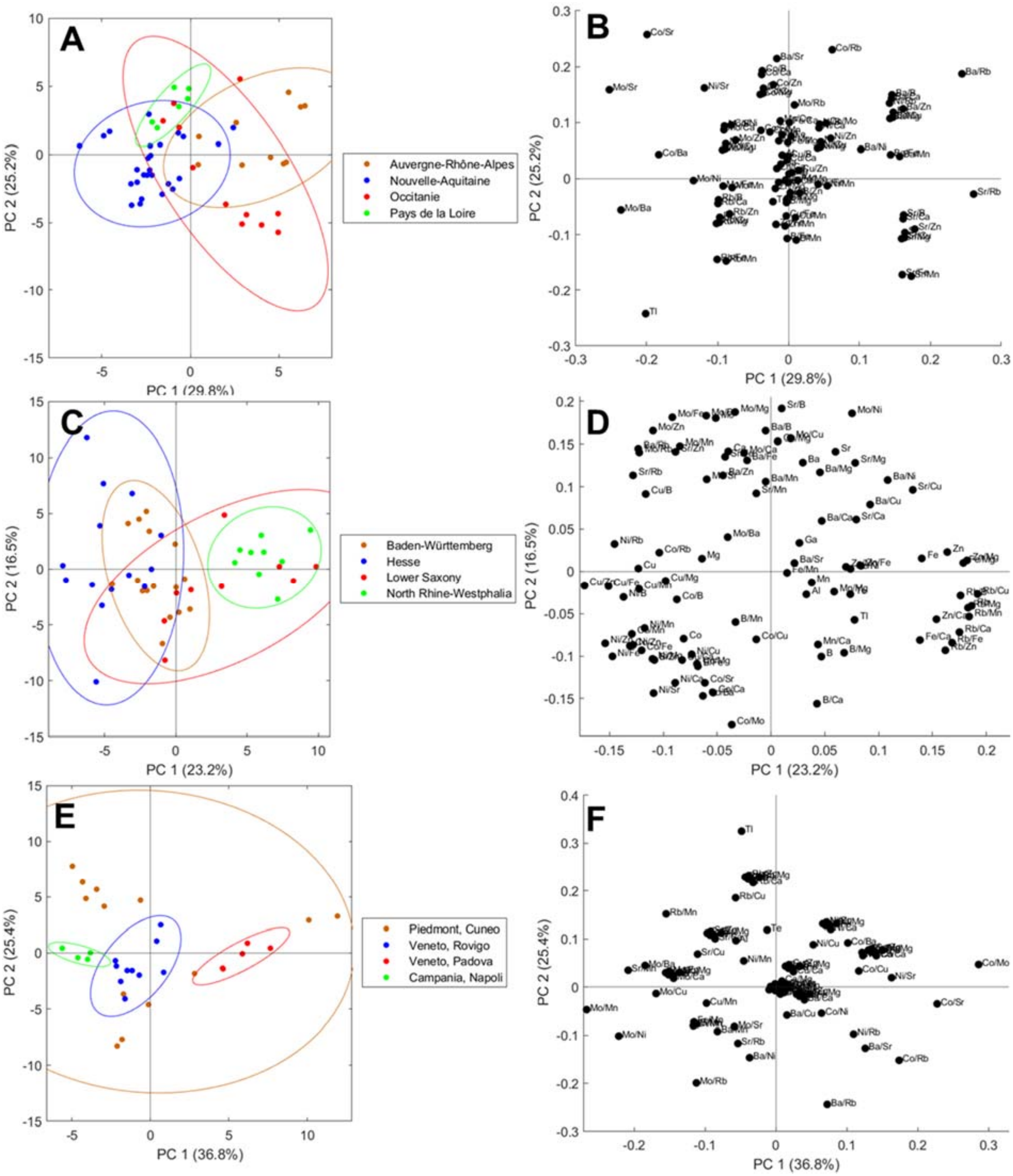

Figure S3: PCA models for the differentiation of walnut samples within France, Germany and Italy. Scores with 95% confidence ellipses are colored by French (A), German (C) and Italian (E) regions and the respective loading plots are shown in (B), (D) and (F).

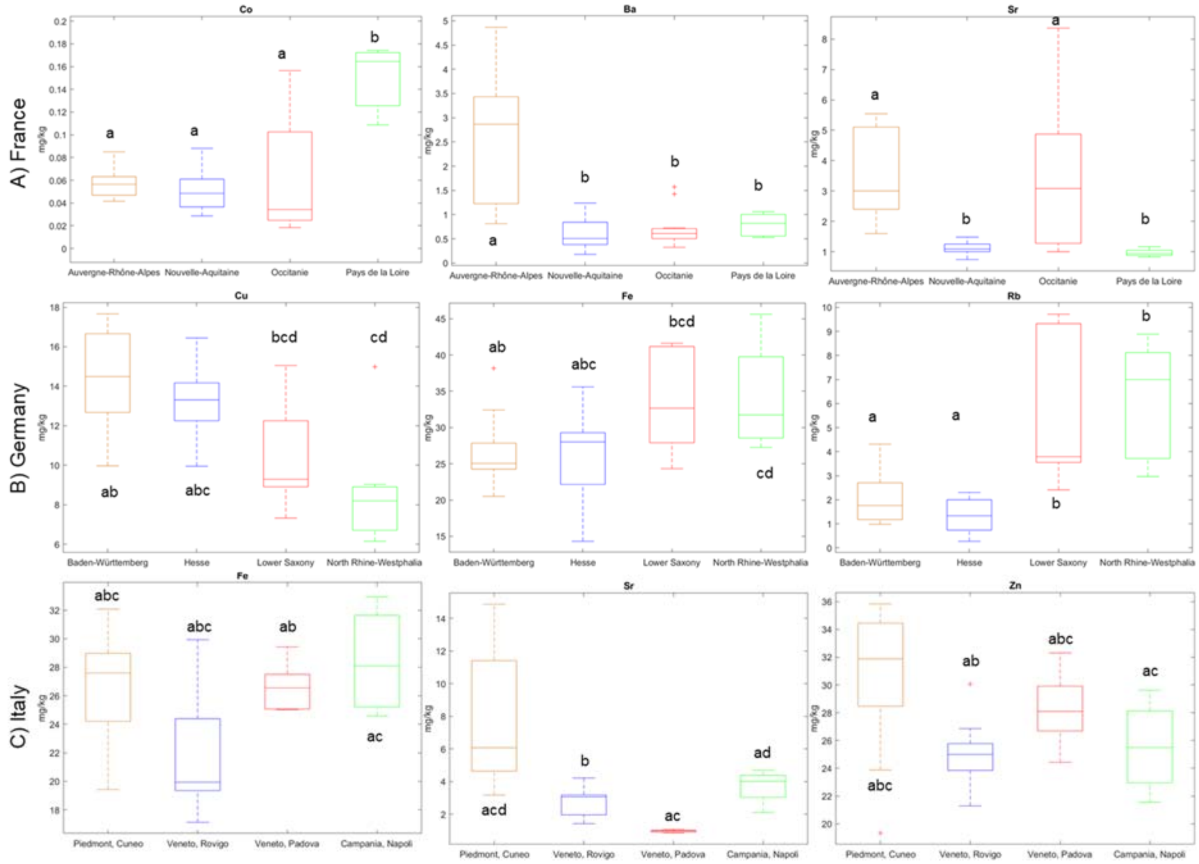

Figure S4: Box-plots for the significant elements for the walnuts' origins authentication on a regional level in France, Germany and Italy after one-way ANOVA testing. Different small letters indicate significant inter-class differences as determined by Bonferroni post-hoc tests. Data expressed as mg/kg in walnut lyophilizate.

Table S1: Reagents and materials used in this study.

categorization	reagents and materials	supplier
gas	Argon (99,999 %)	Heide Gas Aero GmbH (Lüneburg, Germany) and Westfalen Gas Schweiz GmbH (Eiken, Switzerland)
water	Ultrapure water (>18 MΩ)	Direct-Q purifying system (Merck Millipore Inc., Billerica, MA, USA)
chemicals	Nitric acid (HNO3, ROTIPURAN®Supra, 69 %)	Carl Roth GmbH & Co. KG (Karlsruhe, Germany)
	hydrogen peroxide (H2O2, suprapur®, 30 %)	Merck KGaA (Darmstadt, Germany).
analytes	multi-element solution containing 10 mg/L Sc, Y, La, Ce, Pr, Nd, Sm, Eu, Gd, Tb, Dy, Ho, Er, Tm, Yb, Lu, Th	PerkinElmer Inc. (Waltham, MA, USA)
	multi-element solution containing 10 mg/L Li, Na, Mg, Al, K, V, Cr, Mn, Co, Ni, Cu, Ga, Rb, Sr, Mo, Ag, Cd, Te, Ba, Tl, Pb, Bi, U, 100 mg/L Be, B, Fe, Zn, As, Se and 1,000 mg/L Ca	Merck KGaA (Darmstadt, Germany).
internal standards	1 g/L Ge	Inorganic Ventures Inc. (Christiansburg, VA, USA)
	1 g/L Rh	
	1 g/L In	
	1 g/L Re	

Table S2: Analyzed walnut samples with country and harvest year. When available, the region and the cultivar is given.

origin	harvest year	regional origin	cultivar	counter
Switzerland (CH)	2017	Thurgau	Franquette	1
	2018	Thurgau	Lara	2
	2018	Thurgau	Chandler	3
	2018	Thurgau		4
	2018	Thurgau	Fernette	5
	2018	Thurgau	Pedro	6
	2018	Thurgau	Hartley	7
	2018	Thurgau		8
	2018	Thurgau	Fernor	9
	2018	Thurgau		10
	2018	Thurgau		11
	2018	Thurgau		12
	2018	Thurgau		13
	2019	Thurgau		14
	2019	Thurgau		15
	2019	Thurgau	Franquette	16
	2019	Thurgau	Chandler	17

origin	harvest year	regional origin	cultivar	counter
	2019	Thurgau		18
	2019	Thurgau	Vina	19
	2019	Thurgau	Serr	20
	2019	Thurgau		21
	2019	Thurgau		22
	2019	Thurgau		23
	2019	Thurgau		24
	2019	Thurgau		25
	2019	Thurgau		26
	2019	Thurgau		27
	2019	Thurgau		28
	2019	Thurgau		29
	2019	Thurgau	Lara	30
	2019	Thurgau	Fernor	31
Chile (CL)	2017			1
	2017			2
	2017		Chandler	3
	2017		Serr	4
	2018		Serr	5
China (CN)	2017		Tulare	1
	2017	Yunnan		2
	2017	Xinjiang		3
	2017	Yunnan		4
	2018	Shanxi		5
	2018	Xinjiang	Tulare	6
	2018	Xinjiang		7
	2018	Xinjiang	Chandler	8
	2018	Yunnan		9
	2018	Shanxi		10
	2019	Xinjiang		11
	2019	Xinjiang		12
	2019		Chandler	13
	2019			14
	2019	Yunnan		15
Germany (DE)	2017	Baden-Württemberg		1
	2017	Baden-Württemberg		2
	2017	Schleswig-Holstein		3
	2017	Hesse		4
	2017	Baden-Württemberg		5
	2018	Lower Saxony		6
	2018	Lower Saxony		7
	2018	Lower Saxony		8
	2018	Baden-Württemberg		9
	2018	North Rhine-Westphalia		10
	2018	North Rhine-Westphalia		11

origin	harvest year	regional origin	cultivar	counter
	2018	Hesse	Hartley	12
	2018	Hesse		13
	2018	Hesse		14
	2018	Baden-Württemberg		15
	2018	Baden-Württemberg		16
	2018	Baden-Württemberg		17
	2018	Lower Saxony		18
	2018	Hesse	Franquette	19
	2018	North Rhine-Westphalia		20
	2018	Hesse		21
	2018	Baden-Württemberg		22
	2018	Lower Saxony		23
	2018	Lower Saxony		24
	2018	Baden-Württemberg		25
	2018	Baden-Württemberg		26
	2019	Baden-Württemberg		27
	2019	North Rhine-Westphalia		28
	2019	Baden-Württemberg		29
	2019	Saxony-Anhalt		30
	2019	Baden-Württemberg		31
	2019	Lower Saxony		32
	2019	Hesse		33
	2019	Hesse	Lara	34
	2019	Hesse		35
	2019	Hesse		36
	2019	Hesse		37
	2019	Lower Saxony		38
	2019	North Rhine-Westphalia		39
	2019	Hesse		40
	2019	North Rhine-Westphalia		41
	2019	North Rhine-Westphalia		42
	2019	Baden-Württemberg		43
	2019	Baden-Württemberg		44
	2019	North Rhine-Westphalia		45
	2019	North Rhine-Westphalia		46
	2019	Baden-Württemberg		47
	2019	Lower Saxony		48
	2019	Hesse	Lara	49
	2019	Hesse		50
France (FR)	2017	Nouvelle-Aquitaine	Lara	1
	2017	Nouvelle-Aquitaine	Lara	2
	2017	Nouvelle-Aquitaine	Fernor	3
	2017	Occitanie	Lara	4
	2017	Nouvelle-Aquitaine	Fernor	5
	2017		Fernor	6

origin	harvest year	regional origin	cultivar	counter
	2017		Lara	7
	2017	Auvergne-Rhône-Alpes	Franquette	8
	2017	Occitanie	Lara	9
	2017		Franquette	10
	2017		Lara	11
	2017	Pays de la Loire	Franquette	12
	2017	Nouvelle-Aquitaine	Franquette	13
	2017	Auvergne-Rhône-Alpes		14
	2017	Pays de la Loire	Lara	15
	2017	Nouvelle-Aquitaine	Lara	16
	2017	Occitanie	Lara	17
	2017	Nouvelle-Aquitaine	Lara	18
	2017	Auvergne-Rhône-Alpes	Franquette	19
	2017	Occitanie	Lara	20
	2017			21
	2017	Occitanie	Lara	22
	2017	Nouvelle-Aquitaine	Lara	23
	2018	Nouvelle-Aquitaine	Lara	24
	2018	Nouvelle-Aquitaine	Lara	25
	2018	Nouvelle-Aquitaine	Franquette	26
	2018	Nouvelle-Aquitaine	Lara	27
	2018		Franquette	28
	2018	Occitanie	Franquette	29
	2018	Nouvelle-Aquitaine	Franquette	30
	2018	Nouvelle-Aquitaine	Franquette	31
	2018			32
	2018	Auvergne-Rhône-Alpes		33
	2018	Nouvelle-Aquitaine	Fernor	34
	2018	Nouvelle-Aquitaine	Fernor	35
	2018	Nouvelle-Aquitaine	Lara	36
	2018	Occitanie	Lara	37
	2019	Pays de la Loire	Franquette	38
	2019	Auvergne-Rhône-Alpes	Franquette	39
	2019	Occitanie	Franquette	40
	2019	Auvergne-Rhône-Alpes	Lara	41
	2019			42
	2019	Nouvelle-Aquitaine	Fernor	43
	2019			44
	2019	Pays de la Loire	Lara	45
	2019	Occitanie	Franquette	46
	2019	Occitanie	Lara	47
	2019	Nouvelle-Aquitaine	Franquette	48
	2019	Nouvelle-Aquitaine	Fernor	49
	2019	Pays de la Loire	Franquette	50
	2019	Nouvelle-Aquitaine	Franquette	51

origin	harvest year	regional origin	cultivar	counter
	2019	Nouvelle-Aquitaine	Lara	52
	2019	Pays de la Loire	Lara	53
	2019	Auvergne-Rhône-Alpes	Lara	54
	2019	Nouvelle-Aquitaine	Lara	55
	2019	Nouvelle-Aquitaine	Franquette	56
	2019	Auvergne-Rhône-Alpes	Lara	57
	2019	Auvergne-Rhône-Alpes	Lara	58
	2019		Franquette	59
	2019	Nouvelle-Aquitaine	Fernor	60
	2019	Nouvelle-Aquitaine	Lara	61
	2019	Auvergne-Rhône-Alpes	Lara	62
	2019	Occitanie	Lara	63
Hungary (HU)	2017	Tolna		1
	2017			2
	2017	Somogy		3
	2017	Fejér		4
	2017			5
	2017			6
	2018	Baranya		7
	2018	Somogy		8
	2018			9
	2018	Szabolcs-Szatmár-Bereg		10
	2019			11
Italy (IT)	2017	Piedmont	Chandler	1
	2017	Veneto	Lara	2
	2017	Veneto	Lara	3
	2018	Veneto	Tulare	4
	2018	Veneto	Lara	5
	2018	Veneto	Chandler	6
	2018	Veneto	Lara	7
	2018	Piedmont	Chandler	8
	2018	Veneto	Chandler	9
	2018	Campania	Chandler	10
	2018	Piedmont	Tulare	11
	2018	Veneto	Tulare	12
	2018	Veneto	Chandler	13
	2018	Piedmont	Lara	14
	2018	Veneto	Lara	15
	2018	Veneto	Tulare	16
	2018	Piedmont	Lara	17
	2018	Piedmont	Tulare	18
	2019	Piedmont	Lara	19
	2019	Veneto	Tulare	20
	2019	Campania	Tulare	21
	2019	Piedmont	Tulare	22

origin	harvest year	regional origin	cultivar	counter
	2019	Piedmont	Lara	23
	2019	Piedmont	Chandler	24
	2019	Veneto	Chandler	25
	2019	Piedmont	Lara	26
	2019	Piedmont	Lara	27
	2019	Veneto	Lara	28
	2019	Campania	Tulare	29
	2019	Campania	Chandler	30
	2019	Veneto	Chandler	31
	2019	Piedmont	Chandler	32
	2019	Fossano	Lara	33
Pakistan (PK)	2017	Kashmir		1
	2017	Kashmir		2
	2017	Kashmir		3
	2017	Kashmir		4
	2017	Kashmir		5
	2017	Kashmir		6
	2017	Kashmir		7
	2017	Kashmir		8
Turkey (TR)	2017	Kocaeli	Chandler	1
	2017	Kocaeli	Fernor	2
	2017	Kocaeli		3
	2018	Burdur		4
	2018	Kocaeli		5
	2018	Burdur		6
USA	2017	California	Tulare	1
	2017	California		2
	2017	California	Franquette	3
	2017	California	Chandler	4
	2018	California	Hartley	5
	2018	California	Vina	6
	2018		Chandler	7
	2018	California	Serr	8
	2018	California	Chandler	9
	2018	California		10
	2018	California	Howard	11
	2019	Wisconsin		12
	2019		Howard	13
	2019		Tulare	14
	2019	California		15

Table S3: Microwave digestion procedure.

time *t* [min]	temperature T1 [°C] in the teflon vessels	temperature T2 [°C] in the microwave digestion unit
0	23	23
3	85	80
12	145	110
16	200	110
36	200	120
70	23	23
After cooling, the samples were transferred to tubes and filled up to 10 mL with ultrapure water.		

Table S4: Limit of detection (LOD) and limit of quantitation (LOQ) for the measured isotopes for the HR-ICP-MS instrument. Additionally, the respective internal standard element is given.

	**unit**	**LOD**	**LOQ**	**internal standard**
Li7(LR)	[ng/L]	197.7	395.3	Ge72(LR)
Be9(LR)	[ng/L]	3.1	6.2	Ge72(LR)
B11(LR)	[ng/L]	229.6	459.3	Ge72(LR)
Na23(LR)	[µg/L]	0.95	1.90	Ge72(LR)
Mg24(MR)	[ng/L]	216.5	433.0	Ge72(MR)
Al27(MR)	[ng/L]	191.3	382.6	Ge72(MR)
K39(HR)	[µg/L]	1.46	2.92	Ge72(HR)
Ca44(MR)	[ng/L]	116.0	232.1	Ge72(MR)
Sc45(MR)	[ng/L]	0.7	1.5	Ge72(MR)
V51(MR)	[ng/L]	15.7	31.4	Ge72(MR)
Cr52(MR)	[ng/L]	10.9	21.8	Ge72(MR)
Mn55(MR)	[ng/L]	26.9	53.8	Ge72(MR)
Fe56(MR)	[ng/L]	27.6	55.2	Ge72(MR)
Co59(MR)	[ng/L]	2.8	5.6	Ge72(MR)
Ni60(MR)	[ng/L]	41.8	83.6	Ge72(MR)
Cu63(MR)	[ng/L]	17.8	35.6	Ge72(MR)
Zn66(MR)	[ng/L]	178.3	356.7	Ge72(MR)
Ga71(MR)	[ng/L]	3.9	7.8	Ge72(MR)
As75(HR)	[ng/L]	92.1	184.2	Ge72(HR)
Se78(HR)	[µg/L]	1.02	2.04	Ge72(HR)
Rb85(LR)	[ng/L]	8.0	16.0	In115(LR)
Sr88(LR)	[ng/L]	7.4	14.9	In115(LR)
Y89(LR)	[ng/L]	0.5	0.9	Rh103(LR)
Mo95(LR)	[µg/L]	0.64	1.28	Rh103(LR)
Ag109(LR)	[ng/L]	84.0	168.1	Rh103(LR)
Cd111(LR)	[ng/L]	8.5	17.0	Rh103(LR)
Te125(LR)	[ng/L]	13.9	27.9	Rh103(LR)
Ba137(LR)	[ng/L]	32.0	64.0	In115(LR)
La139(LR)	[ng/L]	0.1	0.3	In115(LR)
Ce140(LR)	[ng/L]	0.5	1.0	In115(LR)
Pr141(LR)	[ng/L]	0.1	0.2	In115(LR)
Nd146(LR)	[ng/L]	0.4	0.8	In115(LR)
Sm147(LR)	[ng/L]	0.6	1.3	In115(LR)
Eu153(MR)	[ng/L]	0.5	1.0	In115(MR)
Gd157(LR)	[ng/L]	0.1	0.3	In115(LR)
Tb159(LR)	[ng/L]	0.1	0.2	In115(LR)
Dy163(LR)	[ng/L]	0.2	0.4	In115(LR)
Ho165(LR)	[ng/L]	0.1	0.1	In115(LR)
Er166(LR)	[ng/L]	0.1	0.2	In115(LR)
Tm169(LR)	[ng/L]	0.1	0.1	In115(LR)
Yb172(LR)	[ng/L]	0.1	0.3	In115(LR)
Lu175(LR)	[ng/L]	0.1	0.1	In115(LR)
Tl205(LR)	[ng/L]	0.9	1.8	In115(LR)

	**unit**	**LOD**	**LOQ**	**internal standard**
Pb208(LR)	[ng/L]	2.2	4.5	Re185(LR)
Bi209(LR)	[ng/L]	1.3	2.7	Re185(LR)
Th232(LR)	[ng/L]	0.2	0.4	Re185(LR)
U238(LR)	[ng/L]	2.9	5.7	Re185(LR)

Table S5: Instrumental conditions and measurement parameters for the Element2 HR-ICP-MS instrument.

**forward power (W)**	1225		
**gas flow rates (mL/min)**			
**plasma gas**	16.0		
**auxiliary gas**	0.70		
**nebulizer gas**	1.179		
**sample uptake (μL/min)**	200		
**cones**	Ni		
**nebuliser**	MicroMistTM		
**number of acquisition replica**	3		
**tuning**	doubly charged: Ba++/Ba+, oxide ratio: BaO+/Ba+		
**acquisition mode**	E-Scan		
**resolution**	Low (300 mΔm-1)	Medium (4000 mΔm-1)	High (10,000 mΔm-1)
**selected isotopes for analytes**	7Li, 9Be, 11B, 23Na, 85Rb, 88Sr, 95Mo, 107Ag, 125Te, 137Ba, 139La, 140Ce, 141Pr, 146Nd, 147Sm, 157Gd, 159Tb, 163Dy, 165Ho, 166Er, 169Tm, 172Yb, 175Lu, 205Tl, 208Pb, 209Bi, 232Th, 238U	24Mg, 27Al, 44Ca, 45Sc, 51V, 52Cr, 55Mn, 56Fe, 59Co, 60Ni, 63Cu, 66Zn, 71Ga, 111Cd, 153Eu	39K, 75As, 78Se
**selected isotopes for internal standard**	72Ge, 103Rh, 115In, 185Re	72Ge, 103Rh, 115In	72Ge
**correction equations**	115In=115In–0.0149·Sn118<br>Pb=206Pb+207Pb+208Pb	115In=115In–0.0149·Sn118	78Se=78Se–0.0304·83Kr

Table S6: Mean concentration and standard deviation (sd) for walnut origins in mg/kg.

	CH	CL	CN	DE	FR	HU	IT	PK	TR	US
Al [mg/kg]	0.142 ± 0.17	1.256 ± 1.563	0.533 ± 0.432	0.418 ± 0.359	0.268 ± 0.216	0.855 ± 1.127	0.407 ± 0.391	0.79 ± 0.366	0.337 ± 0.259	0.745 ± 1.175
B [mg/kg]	15.1 ± 2.9	15.7 ± 2	12.7 ± 3.2	13.8 ± 2.4	15.7 ± 2.2	13.1 ± 2.5	15.4 ± 2.9	13.3 ± 2.7	14.5 ± 2.8	13.2 ± 3.2
Ba [mg/kg]	0.945 ± 1.21	1.775 ± 1.117	3.196 ± 3.368	1.207 ± 0.616	1.04 ± 0.938	1.743 ± 1.105	1.593 ± 1.467	0.574 ± 1.305	0.329 ± 0.498	0.323 ± 4.032
Ca [g/kg]	0.908 ± 0.241	1.046 ± 0.189	0.996 ± 0.182	0.911 ± 0.201	0.779 ± 0.191	1.006 ± 0.131	0.969 ± 0.139	0.991 ± 0.182	1.029 ± 0.099	1.004 ± 0.197
Co [mg/kg]	0.039 ± 0.024	0.086 ± 0.034	0.081 ± 0.052	0.044 ± 0.021	0.063 ± 0.039	0.056 ± 0.033	0.093 ± 0.085	0.07 ± 0.024	0.108 ± 0.05	0.08 ± 0.054
Cu [mg/kg]	16.2 ± 3	14.6 ± 2.1	15.9 ± 2.8	12.1 ± 3.4	13.7 ± 2.2	13.1 ± 2.7	16.3 ± 3.5	15.7 ± 3.1	14.1 ± 2.9	13 ± 2.8
Fe [mg/kg]	29.2 ± 4.2	38.1 ± 3.2	30.1 ± 3.3	29.7 ± 6.7	22.2 ± 5.8	28.6 ± 3.5	25.3 ± 4.4	31.2 ± 4.9	29.3 ± 4.2	28.9 ± 5.6
Ga [mg/kg]	0.069 ± 0.012	0.061 ± 0.011	0.068 ± 0.018	0.064 ± 0.018	0.057 ± 0.013	0.067 ± 0.01	0.057 ± 0.013	0.063 ± 0.007	0.067 ± 0.012	0.064 ± 0.011
Mg [g/kg]	1.76 ± 0.16	1.6 ± 0.15	1.89 ± 0.2	1.7 ± 0.26	1.68 ± 0.24	1.75 ± 0.25	1.74 ± 0.23	1.81 ± 0.12	1.76 ± 0.18	1.7 ± 0.19
Mn [mg/kg]	36.3 ± 11.8	43.9 ± 13	66.4 ± 52.5	34.1 ± 16.7	26.8 ± 12.7	36.2 ± 9.9	34.5 ± 55.2	29.7 ± 9	41.1 ± 14.8	37.7 ± 30.4
Mo [mg/kg]	0.227 ± 0.083	0.273 ± 0.109	0.364 ± 0.173	0.269 ± 0.152	0.173 ± 0.127	0.288 ± 0.12	0.407 ± 0.255	0.661 ± 0.135	0.15 ± 0.024	0.253 ± 0.173
Ni [mg/kg]	2.08 ± 0.83	1.32 ± 0.53	2.01 ± 1.21	1.85 ± 0.97	1.94 ± 0.94	3.06 ± 1.53	2.62 ± 1.68	1.62 ± 1.48	3.71 ± 1.47	3.29 ± 1.21
Rb [mg/kg]	4.85 ± 3.64	7.09 ± 4.04	4.31 ± 2.96	3.25 ± 2.58	3.06 ± 1.74	4.5 ± 2.66	5.7 ± 5.13	3.84 ± 2.32	2.7 ± 0.87	6.29 ± 3.74
Sr [mg/kg]	2.22 ± 1.21	6.27 ± 1.91	11.37 ± 6.52	2.28 ± 1.01	2.07 ± 1.65	3.02 ± 1.64	4.73 ± 3.8	4.09 ± 1.6	3.39 ± 1.63	9.81 ± 4.59
Te [µg/kg]	0.4 ± 0.157	0.615 ± 0.154	0.407 ± 0.239	0.65 ± 0.461	0.78 ± 0.555	0.397 ± 0.281	0.96 ± 0.78	0.371 ± 0.178	0.452 ± 0.135	0.456 ± 0.148
Tl [µg/kg]	1.152 ± 1.1	3.346 ± 1.887	0.815 ± 1.165	1.331 ± 2.588	1.437 ± 1.763	1.977 ± 1.514	4.68 ± 4.882	1.135 ± 1.431	1.278 ± 0.586	2.264 ± 1.651
Zn [mg/kg]	32 ± 5.5	28.6 ± 1.8	24 ± 4.5	28.4 ± 9	23.1 ± 3.3	26.5 ± 3.7	27.8 ± 4.5	24.1 ± 5	26.6 ± 4.5	28.1 ± 4.9

Table S7: Overall accuracy with standard deviation (in %) for different data pre-treatment and classification methods for the predictions of all walnut samples using stratified nested cross validation. The standard deviation was calculated using the 20 repetitions of the entire cross validation process.

		**classification method**			
		1) LDA	2) SVM	3) SSD	4) RF
**data pre-treatment**	i) no data-pretreatment	61.4 ± 2.0	72.6 ± 1.3	68.5 ± 1.5	50.1 ± 2.4
	ii) log10	60.6 ± 2.1	72.7 ± 1.1	60.9 ± 1.2	50.9 ± 3.9
	iii) center (mean), scale (standard deviation)	61.8 ± 2.6	72.9 ± 1.6	67.9 ± 1.7	49.8 ± 2.7
	iv) center (median), scale (standard deviation)	61.4 ± 2.1	72.6 ± 1.4	69.2 ± 1.4	49.8 ± 3.2
	v) center (median), scale (range)	61.9 ± 2.3	72.4 ± 1.3	68.6 ± 1.4	50.0 ± 3.1
	vi) center (median), scale (interquartile range)	62.1 ± 2.0	72.3 ± 1.4	68.1 ± 2.1	50.1 ± 3.4

Table S8: Overall accuracy (in %) for different data pre-treatment and classification methods for the predictions of all walnut samples using leave-one-out-cross validation.

		**classification method**			
		1) LDA	2) SVM	3) SSD	4) RF
**data pre-treatment**	i) no data-pre-treatment	65.8	75.5	69.6	49.8
	ii) log10	62.9	74.3	60.8	49.8
	iii) center (mean), scale (standard deviation)	65.8	75.5	67.5	49.8
	iv) center (median), scale (standard deviation)	65.8	75.5	68.4	49.8
	v) center (median), scale (range)	65.8	75.5	70.0	49.8
	vi) center (median), scale (interquartile range)	65.8	75.5	67.9	49.8

Table S9: Mean concentration and standard deviation for walnut samples from French regions.

	**Auvergne-Rhône-Alpes**	**Nouvelle-Aquitaine**	**Occitanie**	**Pays de la Loire**
Al [mg/kg]	0.18 ± 0.078	0.258 ± 0.207	0.224 ± 0.193	0.182 ± 0.075
B [mg/kg]	15.2 ± 1.8	15.8 ± 2.0	17.0 ± 2.6	12.9 ± 1.6
Ba [mg/kg]	2.59 ± 1.39	0.59 ± 0.29	0.72 ± 0.41	0.8 ± 0.24
Ca [g/kg]	0.8 ± 0.18	0.78 ± 0.21	0.77 ± 0.14	0.64 ± 0.09
Co [mg/kg]	0.058 ± 0.013	0.051 ± 0.017	0.063 ± 0.049	0.152 ± 0.028
Cu [mg/kg]	13.7 ± 1.4	13.5 ± 2.7	13.8 ± 2.3	13.3 ± 2.0
Fe [mg/kg]	25.8 ± 3.5	20.0 ± 5.8	19.4 ± 6.7	25.6 ± 2.2
Ga [mg/kg]	0.06 ± 0.01	0.059 ± 0.015	0.05 ± 0.012	0.063 ± 0.013
Mg [g/kg]	1.8 ± 0.3	1.6 ± 0.2	1.69 ± 0.23	1.76 ± 0.17
Mn [mg/kg]	28.6 ± 5.0	22.1 ± 6.8	21.2 ± 6.9	37.4 ± 4.9
Mo [mg/kg]	0.165 ± 0.052	0.214 ± 0.177	0.125 ± 0.075	0.102 ± 0.018
Ni [mg/kg]	3.18 ± 1.12	1.63 ± 0.57	1.38 ± 0.74	1.84 ± 0.61
Rb [mg/kg]	3.39 ± 2.71	4.03 ± 1.29	2.10 ± 1.24	2.57 ± 0.61
Sr [mg/kg]	3.39 ± 1.45	1.11 ± 0.2	3.51 ± 2.44	0.96 ± 0.13
Te [µg/kg]	0.621 ± 0.478	0.772 ± 0.571	1.182 ± 0.463	0.76 ± 0.63
Tl [µg/kg]	2.35 ± 3.33	1.73 ± 1.4	0.99 ± 1.04	1.34 ± 1.16
Zn [mg/kg]	22.5 ± 1.9	23.8 ± 3.2	21.9 ± 3.3	20.1 ± 2.4

Table S10: Mean concentration and standard deviation for walnut samples from German regions.

	**Baden-Württemberg**	**Hesse**	**Lower Saxony**	**North Rhine-Westphalia**
Al [mg/kg]	0.515 ± 0.307	0.265 ± 0.230	0.289 ± 0.256	0.651 ± 0.557
B [mg/kg]	14.5 ± 1.6	12.3 ± 3.2	13.8 ± 1.7	14.5 ± 2.6
Ba [mg/kg]	1.16 ± 0.59	0.95 ± 0.43	1.21 ± 0.79	1.43 ± 0.49
Ca [g/kg]	0.87 ± 0.16	1.03 ± 0.21	0.82 ± 0.26	0.89 ± 0.17
Co [mg/kg]	0.046 ± 0.012	0.049 ± 0.027	0.052 ± 0.029	0.027 ± 0.009
Cu [mg/kg]	14.5 ± 2.4	13.0 ± 1.9	10.4 ± 2.7	8.5 ± 2.7
Fe [mg/kg]	26.4 ± 4.3	26.5 ± 5.6	34.1 ± 6.8	34.3 ± 6.6
Ga [mg/kg]	0.063 ± 0.012	0.06 ± 0.008	0.067 ± 0.018	0.061 ± 0.017
Mg [g/kg]	1.66 ± 0.23	1.81 ± 0.3	1.72 ± 0.12	1.54 ± 0.22
Mn [mg/kg]	27.9 ± 9.1	31.6 ± 13.9	45.5 ± 26.7	33.6 ± 12.6
Mo [mg/kg]	0.203 ± 0.11	0.365 ± 0.21	0.254 ± 0.072	0.225 ± 0.087
Ni [mg/kg]	1.69 ± 0.44	2.65 ± 1.11	1.7 ± 1.15	1.1 ± 0.56
Rb [mg/kg]	2.09 ± 1.04	1.32 ± 0.69	5.5 ± 3.03	6.06 ± 2.29
Sr [mg/kg]	2.29 ± 0.64	2.0 ± 0.36	2.04 ± 0.83	2.5 ± 1.12
Te [µg/kg]	0.573 ± 0.45	0.478 ± 0.363	0.93 ± 0.541	0.828 ± 0.48
Tl [µg/kg]	0.25 ± 0.34	0.44 ± 0.35	3.16 ± 4.07	3.01 ± 3.71
Zn [mg/kg]	25.3 ± 4.2	22.0 ± 2.6	35.7 ± 13.1	34.1 ± 8.6

Table S11: Mean concentration and standard deviation for walnut samples from Italian regions.

	**Campania, Napoli**	**Piedmont, Cuneo**	**Veneto, Padova**	**Veneto, Rovigo**
Al [mg/kg]	0.45 ± 0.111	0.478 ± 0.499	0.309 ± 0.333	0.386 ± 0.357
B [mg/kg]	16.2 ± 2.6	13.9 ± 2.4	16.0 ± 2.0	16.6 ± 3.3
Ba [mg/kg]	0.92 ± 0.15	2.05 ± 2.13	0.66 ± 0.08	1.71 ± 0.77
Ca [g/kg]	0.95 ± 0.09	1.02 ± 0.17	0.97 ± 0.06	0.94 ± 0.13
Co [mg/kg]	0.031 ± 0.012	0.12 ± 0.11	0.162 ± 0.057	0.047 ± 0.02
Cu [mg/kg]	14.1 ± 3.5	15.8 ± 4.1	19.0 ± 1.8	16.6 ± 3.1
Fe [mg/kg]	28.4 ± 3.9	26.6 ± 4.0	26.6 ± 1.8	21.5 ± 4.2
Ga [mg/kg]	0.052 ± 0.007	0.062 ± 0.015	0.051 ± 0.014	0.056 ± 0.012
Mg [g/kg]	1.67 ± 0.28	1.75 ± 0.24	1.63 ± 0.23	1.81 ± 0.2
Mn [mg/kg]	14.7 ± 3.3	57 ± 83.9	31.2 ± 12.1	14.8 ± 3.6
Mo [mg/kg]	0.483 ± 0.104	0.517 ± 0.327	0.131 ± 0.014	0.395 ± 0.123
Ni [mg/kg]	0.88 ± 0.65	3.27 ± 2.25	2.44 ± 0.37	2.65 ± 1.02
Rb [mg/kg]	6.08 ± 0.85	8.24 ± 7.25	3.7 ± 1.2	3.66 ± 2.16
Sr [mg/kg]	3.71 ± 1.12	7.89 ± 4.18	0.96 ± 0.08	2.87 ± 0.92
Te [μg/kg]	0.99 ± 0.411	1.116 ± 0.934	0.823 ± 0.691	0.76 ± 0.781
Tl [μg/kg]	9.15 ± 2.49	6.13 ± 6.57	2.07 ± 0.61	2.73 ± 1.77
Zn [mg/kg]	25.5 ± 3.4	30.8 ± 5.0	28.3 ± 2.8	25.1 ± 2.3

Table S12: Overall accuracy with standard deviation (in %) for different data pre-treatment and classification methods for the predictions of French walnuts for a regional differentiation in France using stratified nested cross validation. The standard deviation was calculated using the 20 repetitions of the entire cross validation process.

		**classification method**			
		1) LDA	2) SVM	3) SSD	4) RF
**data pre-treatment**	i) no data-pretreatment	86.2 ± 3.3	90.1 ± 2.4	87.1 ± 2.7	79.6 ± 5.4
	ii) log10	82.4 ± 4.6	91.4 ± 2.1	88.4 ± 3.2	81.0 ± 3.7
	iii) center (mean), scale (standard deviation)	86.9 ± 3.0	90.1 ± 2.4	89.2 ± 3.7	80.3 ± 4.9
	iv) center (median), scale (standard deviation)	84.5 ± 4.4	89.9 ± 3.3	87.1 ± 2.7	80.2 ± 4.3
	v) center (median), scale (range)	86.3 ± 2.6	90.8 ± 2.9	88.7 ± 2.8	81.6 ± 3.0
	vi) center (median), scale (interquartile range)	86.0 ± 3.7	90.5 ± 2.9	88.8 ± 4.3	79.6 ± 3.4

Table S13: Overall accuracy with standard deviation (in %) for different data pre-treatment and classification methods for the predictions of German walnuts for a regional differentiation in Germany using nested cross validation. The standard deviation was calculated using the 20 repetitions of the entire cross validation process.

		**classification method**			
		1) LDA	2) SVM	3) SSD	4) RF
**data pre-treatment**	i) no data-pretreatment	58.5 ± 3.8	76.7 ± 3.3	75.2 ± 3.6	65.3 ± 5.1
	ii) log10	68.6 ± 3.4	74.5 ± 1.8	72.3 ± 4.2	60.5 ± 6.3
	iii) center (mean), scale (standard deviation)	60.0 ± 4.2	76.8 ± 3.0	76.1 ± 3.9	63.1 ± 3.4
	iv) center (median), scale (standard deviation)	62.3 ± 5.7	77.4 ± 2.5	75 ± 3.6	61.9 ± 6.7
	v) center (median), scale (range)	61.5 ± 4.9	76.6 ± 2.9	75.2 ± 2.4	63.4 ± 5.9
	vi) center (median), scale (interquartile range)	59.9 ± 5.5	76.3 ± 2.8	74.3 ± 4.1	62.2 ± 6.7

Table S14: Overall accuracy with standard deviation (in %) for different data pre-treatment and classification methods for the predictions of Italian walnuts for a regional differentiation in Italy using nested cross validation. The standard deviation was calculated using the 20 repetitions of the entire cross validation process.

		**classification method**			
		1) LDA	2) SVM	3) SSD	4) RF
**data pre-treatment**	i) no data-pretreatment	82.2 ± 3.6	91.7 ± 2.6	81.9 ± 3.9	60.6 ± 6.1
	ii) log10	70.2 ± 8.7	94.2 ± 2.8	83.8 ± 4.7	61.9 ± 4
	iii) center (mean), scale (standard deviation)	79.8 ± 5.1	92.8 ± 2.6	82 ± 4.2	61.6 ± 3.2
	iv) center (median), scale (standard deviation)	79.1 ± 5.1	92.3 ± 3.2	82.5 ± 5.4	60.2 ± 6.1
	v) center (median), scale (range)	79.1 ± 5.9	93.1 ± 2.4	81.6 ± 6.2	61.3 ± 5.7
	vi) center (median), scale (interquartile range)	78.9 ± 6.3	91.7 ± 2.8	83 ± 5.3	57.8 ± 5.4

Table S15. Overall accuracies of 1-vs-1 binary classification using stratified nested cross-validation of 20 repetitions (classification method: quadratic SVM, data pre-treatment: log10 transformation).

**vs.**	CH	CL	CN	DE	FR	HU	IT	PK	TR	US
CH	-	95.4	97.1	83.2	89.3	92.4	94.5	98.6	90.8	91.4
CL		-	93.0	98.2	98.4	98.1	93.7	98.5	85.9	84.8
CN			-	99.5	99.9	92.7	97.0	90.0	98.1	93.3
DE				-	85.1	87.8	93.0	96.7	94.9	96.6
FR					-	94.4	95.9	99.7	95.5	92.4
HU						-	93.6	90.5	84.7	88.5
IT							-	93.3	89.9	90.8
PK								-	98.9	89.6
TR									-	81.7
US										-
